# LAP3 contributes to IFN-γ-induced arginine depletion and malignant transformation of bovine mammary epithelial cells

**DOI:** 10.1186/s12885-022-09963-w

**Published:** 2022-08-08

**Authors:** Li Li, Fengyang Li, Xiuhong Hu, Zengshuai Wu, Wenbo Ren, Tingting Wang, Zhengchao Ji, Na Li, Jingmin Gu, Changjiang Sun, Xin Feng, Wenyu Han, Jing Huang, Liancheng Lei

**Affiliations:** 1 Department of First Hospital, Jilin University, Xinmin Street 1, Changchun, China; 2 State Key Laboratory for Zoonotic Diseases, College of Veterinary Medicine, Jilin University, Xi’an Road 5333, Changchun, China; 3 Shannan Hospital, Shannan, China

**Keywords:** IFN-γ, Arginine depletion, Malignant transformation, LAP3, ASS1, HDAC2

## Abstract

**Background:**

IFN-γ has been traditionally recognized as an inflammatory cytokine that involves in inflammation and autoimmune diseases. Previously we have shown that sustained IFN-γ induced malignant transformation of bovine mammary epithelial cells (BMECs) via arginine depletion. However, the molecular mechanism underlying this is still unknown.

**Methods:**

In this study, the amino acids contents in BMECs were quantified by a targeted metabolomics method. The acquisition of differentially expressed genes was mined from RNA-seq dataset and analyzed bioinformatically. Quantitative reverse transcription polymerase chain reaction (qRT-PCR), enzyme-linked immunosorbent assay (ELISA), western blotting, and immunohistochemistry (IHC) assay were performed to detect gene mRNA and protein expression levels. CCK-8 and would healing assays were used to detect cell proliferation and migration abilities, respectively. Cell cycle phase alternations were analyzed by flow cytometry.

**Results:**

The targeted metabolomics analysis specifically discovered IFN-γ induced arginine depletion through accelerating arginine catabolism and inhibiting arginine anabolism in BMECs. Transcriptome analysis identified leucine aminopeptidase 3 (LAP3), which was regulated by p38 and ERK MAPKs, to downregulate arginine level through interfering with argininosuccinate synthetase (ASS1) as IFN-γ stimulated. Moreover, LAP3 also contributed to IFN-γ-induced malignant transformation of BMECs by upregulation of HDAC2 (histone deacetylase 2) expression and promotion of cell cycle proteins cyclin A1 and D1 expressions. Arginine supplementation did not affect LAP3 and HDAC2 expressions, but slowed down cell cycle process of malignant BMECs. In clinical samples of patients with breast cancer, LAP3 was confirmed to be upregulated, while ASS1 was downregulated compared with healthy control.

**Conclusions:**

These results demonstrated that LAP3 mediated IFN-γ-induced arginine depletion to malignant transformation of BMECs. Our findings provide a potential therapeutic target for breast cancer both in humans and dairy cows.

**Supplementary Information:**

The online version contains supplementary material available at 10.1186/s12885-022-09963-w.

## Introduction

Mammary gland, which produces milk to feed young offspring, is an important organ of mammals. Mammary gland diseases such as mastitis and breast cancer are major threat to both animals and humans, leading to enormous economic losses and numerous death each year. Thus, a better understanding of the regulation of mammary gland facilitates the development of novel therapeutics and prevention of mammary gland diseases.

A number of factors, including dietary, hormonal, environmental and management, influence the health status of mammary gland [1, 2]. A balanced dietary nutrition contributes to maintain normal functions of host mammary gland and resistance mechanisms against infection. While overnutrition can induce systemic metabolic diseases, chronic inflammation and certain types of cancer in humans [3], malnutrition can lead to immune suppression and high incidence of mammary gland diseases [4, 5]. Feeding of dairy cows with corn straw increased the release of endotoxin and proinflammatory cytokines in the mammary gland, leading to decreased milk quality and a high incidence of subacute ruminal acidosis and mastitis [6–9]. Among these cytokines is IFN-γ, a pleiotropic proinflammatory cytokine that is mainly secreted by T cells and natural killer (NK) cells. Abnormal expression of IFN-γ is often associated with inflammation and autoimmune diseases. In animals, elevated IFN-γ level induces metabolic disorders of certain amino acids and subacute inflammation [10]. IFN-γ increases susceptibility to *Staphylococcus aureus* (*S. aureus*) of bovine mammary epithelial cells (BMECs) and eventually causes mastitis of bovine mammary gland [11]. Interestingly, addition of arginine could dramatically alleviate this phenomenon [11], indicating a potential regulatory role of IFN-γ on arginine metabolism of BMECs.

Arginine is one of host semi-essential amino acids for various cellular functions, including cell proliferation and immune responses [12]. Arginine metabolism is tightly regulated and studies have found that abnormal arginine metabolism is closely associated with the development of many diseases, such as cancer and infection [13]. As a pleiotropic cytokine, IFN-γ has both anti- and protumorigenic effects dependent on the cellular, microenvironmental, and/or molecular context [14]. In the context of breast cancer, studies have shown that the antitumor effect of IFN-γ varied dramatically among different breast cancer cell lines and sustained low-level of IFN-γ exposure promoted the development of MA782/5S mammary adenocarcinoma [15, 16]. Arginine is also regulated by IFN-γ to affect malignant cell transformation of BMECs [17], a process associated with carcinogenesis. Compared to normal cells, malignant BMECs displayed a dramatical cell morphology change and accelerations in cell migration and proliferation ability, which could be a suitable model for breast cancer research. Besides, the malignant BMECs showed impaired milk protein and fat synthesis abilities compared to normal cells [2]. However, the detailed molecular mechanism for IFN-γ-induced arginine depletion and malignant transformation of BMECs is still unknown.

Leucine aminopeptidase 3 (LAP3) is identified as a cytosol metallopeptidase protein both in mammals and plants that belongs to the M17 aminopeptidase family. It plays a key role in protein degradation and peptide metabolism through the removal of unsubstituted N-terminal hydrophobic amino acid leucine [18–20]. Recently, studies have found that LAP3 is involved in the regulation of cell proliferation, angiogenesis, and malignant development of various tumor types including ovarian cancer, glioma, esophageal squamous cell carcinoma, liver cancer, lung cancer and breast cancer [21–25]. However, whether LAP3 participates in and the exact molecular mechanism of LAP3 in IFN-γ-induced arginine depletion and malignant transformation of BMECs remains unknown. Interestingly, a recent research showed that LAP3 gene expression was significantly upregulated along with other inflammatory cytokines and/or chemokines following SARS-CoV-2 infection, indicating a potential role of LAP3 in regulating inflammation [26].

In this study, we demonstrated that IFN-γ induced arginine depletion and malignant transformation through upregulation of LAP3 in BMECs. LAP3 inhibited the expression of argininosuccinate synthetase (ASS1) to deplete intracellular arginine levels, while promoted cell malignant transformation through HDAC2 (histone deacetylase 2)-mediated upregulation of G1/S cell cycle transition. Our study demonstrated the molecule mechanism for IFN-γ-induced arginine depletion and highlighted the regulatory role of LAP3 in malignant transformation of BMECs. LAP3 might be a potential therapeutic target for the treatment of breast cancer.

## Materials and methods

### Reagents and antibodies

The antibodies used in this study were specific for GAPDH (ABclonal, Cat# A19056, RRID: *AB_2862549*), LAP3 (Affinity Biosciences, Cat# DF12651, RRID: *AB_2845613*), ASS1 (Affinity Biosciences, Cat# BF0242, RRID: *AB_2833984*), HDAC2 (Cell Signaling Technology, Cat# 5113, RRID: *AB_10624871*), Cyclin D1 (Proteintech, Cat# 60186–1-Ig, RRID: *AB_10793718*), p27 (Proteintech, Cat# 25614–1-AP, RRID: *AB_2880161*), Cyclin A1 (Bioss, bs-22318R, RRID: *AB_2904213*), p38 (Affinity Biosciences, Cat# BF8015), ERK1/2 (Affinity Biosciences, Cat# BF8004, RRID: *AB_2846228*), p-p38 (Affinity Biosciences, Cat# AF4001, RRID: *AB_2835330*), p-ERK1/2 (Affinity Biosciences, Cat# AF1015, RRID: *AB_2834432*). Horseradish peroxidase (HRP)-conjugated goat anti-mouse secondary antibody was purchased from ABclonal (Cat# AS003, RRID: *AB_2769851*). HRP-conjugated goat anti-rabbit secondary antibody was purchased from ABclonal (Cat# AS014, RRID: *AB_2769854*). Bovine IFN-γ was purchased from the Kingfisher Biotech (S. Paul, MN, USA). Valproate (VPA) were purchased from MedChemExpress (Shanghai, China). LAP3 inhibitor bestatin was purchased from APExBIO (Shanghai, China). Bovine leucine aminopeptidase (LAP) ELISA kit (Jianglai, Shanghai), human LAP3 ELISA kit and human ASS1 ELISA kit were purchased from FineTest (Wuhan, China). P38 signaling inhibitor SB203580 and ERK signaling inhibitor PD98059 were purchased from Selleck Chemicals (Shanghai, China). LC–MS grade methanol and formic acid (98%) were bought from Sigma-Aldrich (St. Louis, MO, USA); acetonitrile was bought from Thermo (Shanghai, China). Ultrapure water was obtained with a Milli-Q system (Millipore Co., MA, USA). The isotope-labeled amino acid was bought from Cambridge Isotope Laboratories (Andover, MA, USA). All standards used for targeted metabolomics detection were purchased from Sigma (St. Louis, MO, USA) and all isotope internal standards were purchased from CMASS.

### Cell culture and treatment

The BMECs cell line of MAC-T [27] (provided by Prof. Guoqiang Zhu, Yangzhou University, Yangzhou, China) was used in this study. All the cells were grown in Dulbecco’s modified Eagle’s medium/nutrient mixture F-12 (DMEM/F12) with 10% fetal bovine serum (FBS, CLARK, China), with 100 U/mL penicillin, 100 mg/mL streptomycin and incubated at 37 °C in a humidified atmosphere with 5% CO_2_. When the cells entered the logarithmic growth phase, they were digested using 0.25% trypsin. The cultivation of malignant BMECs was performed according to a previous paper [17]. Briefly, cells were treated with 10 ng/mL IFN-γ and sub-cultured every 3 days (3 days/cycle), and the treatments were repeated for a total of 12 weeks.

### Targeted metabolomics

Targeted metabolomics was performed as previously reported [28, 29]. For sample preparation for quality control (QC) and isotope internal (IS) standards, nine metabolite standards and the IS were dissolved with 90% acetonitrile to a final concentration of 1 mg/mL. Then, 25 μL arginine, 5 μL agmatine, 25 μL creatine, 50 μL glutamic acid, 600 μL putrescine, 50 μL spermine, 400 μL ornithine, 200 μL glutamine and 50 μL citrulline were mixed thoroughly with 3595 μL 90% acetonitrile. The concentrations of the standards arginine, agmatine, creatine, glutamic acid, putrescine, spermine, ornithine, glutamine and citrulline were 5 μg/mL, 1 μg/mL, 5 μg/mL, 10 μg/mL, 120 μg/mL, 10 μg/mL, 80 μg/mL, 40 μg/mL and 10 μg/mL, respectively, in the mixture. Finally, 500 μL of the mixture were added into 4.5 mL pure water to obtain different concentrations of standards solutions. For sample preparation for targeted metabolomics, a proper amount of ethyl water (acetonitrile: water = 1:1, v/v) was used to dilute the internal standard stock solution to a final concentration of 10 μg/mL as the internal standard working solution. Then, 50 μL samples was mixed with 50 μL internal standard working solution, 50 μL ethyl water (acetonitrile: water = 1:1, v/v), and 350 μL acetonitrile containing 1% formic acid. The mixture was vortex-mixed and centrifuged for 10 min at 4 °C, 12,000 rpm. The supernatant was collected for further analysis.

Liquid chromatography was performed with ExionL CAD UPLC equipped with a TripleTOF 5600 MS system. The samples were separated by a ZORBAX Eclipse XDB-C8 column (4.6 × 150 mm, 5 μm) at 30 °C, the injection volume was 15.0 μL. Mobile phase A: 0.1% formic acid; mobile phase B: 95% acetonitrile. Samples elution was performed at a flow rate of 0.2 mL/min. Gradient elution procedure was as follows: 0 – 2.5 min, 2% B; 2.5 – 4.0 min, 2% – 50% B; 4.0 – 6.0 min, 50% B; 6.0 – 6.1 min, 50% – 5% B; 6.1 min – 9.9 min, 5% B; 9.9 min – 10.0 min, 2% B. Samples were detected by electrospray ion source in positive ion mode. Samples were scanned with following parameters: DP = 60 V, CE = 10 eV, 50 – 250 Da, curtain gas (CUR) = 30 psi, atomizing gas (GS1) = 50 psi, heating gas (GS2) = 50 psi, ion spray voltage (ISVF) = 5500 V, ion source temperature = 500 °C. Each group has twelve replicates. Differences between mean values were assessed by two-tailed Student’s *t*-test. *p* < 0.05 was considered statistically significant, which was indicated by "*". *p* < 0.01 indicated that the difference was extremely significant, which was indicated by "**".

### LAP3 expression plasmid construction and transient transfection

The short hairpin RNA (shRNA) on LAP3 (Gene: NM174098) and sh-negative control (NC) were designed and synthesized by PPL (Public Protein/Plasmid Library, China). The sequence of sh-LAP3 is: 5’- GCTCGGGCTCTATGAGTATGA-3’, and the sequence of sh-NC is: 5’-GTTCTCCGAACGTGTCACGTT-3’. The overexpression plasmid of LAP3, pLenti-CMV-LAP3-GFP-Puro, was also designed by PPL (Public Protein/Plasmid Library, China). Cell transient transfection experiments were performed using X-tremeGENE HP DNA Transfection Reagent (Roche, USA). In brief, cells were inoculated in a 6-well plate or a 96-well plate at 37 °C in a humidified atmosphere with 5% CO_2_. The plasmid was diluted with serum-free medium to form a DNA-lipid complex. When the cells reached 70% – 80% confluence, the DNA-lipid complexes were transfected transiently into the cells. After 24 h, the cell samples were used for subsequent experiments.

### Cell viability assay

Different groups of cells were inoculated in a 96-well plate at a density of 3 × 10^3^ cells/well in a total volume of 100 μL for 24 h, and then incubated for 0, 24, 48, and 96 h. 10 μL Cell Counting Kit (CCK)-8 (ELabscience, China) solution was added to each well for another 2 h. The absorbance was measured at 450 nm with a microplate reader. Each group contained three duplicates and a blank (culture medium only). The experiment was carried out three times.

### RNA isolation and qRT-PCR

Cells were treated with IFN-γ in a 6-well plate in different groups for 24 h, and then were washed twice with phosphate buffered saline (PBS). The total RNA was extracted using TRIzol reagent (Invitrogen, USA) according to the manufacturer’s instructions. The RNA concentration was measured by NanoDrop 2000 system (Thermo Scientific). Reverse transcription was performed using 1 μg RNA in each sample by the Prime Script™ RT Master Mix Kit (TaKaRa, Dalian, China). qRT-PCR analysis was performed with the TB Green PCR Master Mix (TaKaRa, Dalian, China). The primers for qRT-PCR analysis are listed in Table S2. The 2^−ΔΔCt^ method was used to determine the relative expression level of each gene [30]. *β-actin* was used as a house-keeping gene for internal normalization. The experiments were performed at least three times, and the data were statistically analyzed.

### ELISA assay

Cells from each group were collected and lysed by 200 μL of RIPA lysis buffer (Beyotime, China) containing 1% protease inhibitor (Solarbio, China). The cell-free supernatant was obtained by centrifugation at 12,000 g, 4 °C for 10 min. The intracellular LAP3 content and the human plasma LAP3 content were determined by ELISA according to the manufacturer’s instructions.

### Western blotting assay

Cells from different groups were harvested and washed twice with PBS, then added the RIPA lysis buffer (Solarbio, China) with 1% protease inhibitor (Solarbio, China). The protein concentration was determined by the BCA method (Thermo, USA) and was adjusted to the same total protein concentration. The proteins were separated using SDS-PAGE (4% stacking and 12% resolving gel) by electrophoresis. After the separation of the proteins, the gel was transferred to the polyvinylidene fluoride membrane and sealed in 5% BSA (or 5% skimmed milk powder) for 90 min. PBST was used as the antibody diluent. The diluted primary antibody (1:1000 (v/v)) was incubated with the membrane overnight at 4 °C, and then washed 3 times in PBST (15 min/wash). Then the membrane was incubated with a 1:4000 (v/v) diluted goat anti-rabbit antibody (Abclonal, China) for 50 min at room temperature and washed 3 times in PBST (15 min/wash). The signals were visualized using the Chemiluminescent HRP Substrate (ECL, Burlington, MA, USA). The relative expression levels of the target proteins were estimated using densitometry (ImageJ [31]). GAPDH was used as an internal control.

### LAP enzymatic activity detection

The experiment was performed in accordance with users’ instructions of Leucine Aminopeptidase (LAP) activity detection kit (Solarbio, China). Briefly, the cells were crushed by ultrasound and centrifuged at 10,000 g, 4 °C for 10 min. The supernatant was collected for enzyme activity detection. Each group of samples have three replicates. The calculation formula of enzyme activity (the optical diameter of the cuvette was 1 cm) was as follows: *LAP (U/10*^*4*^* cell)* = *[ΔA* × *V*_*total*_* / (ε* × *d)* × *10*^*9*^*] / (500* × *V*_*sample*_* / V*_*I*_*) / T* = *1.35* × *ΔA*. V_total_: total volume of reaction; ε: molar extinction coefficient of p-nitroaniline, 9.87 × 10^3^ L/mol/cm; 10^9^: 1 mol = 10^9^ nmol; d: diameters of cuvette, 0.6 cm; V_Sample_: sample volume; V_I_: addition volume of reagent I; T: reaction time, 3 min.

### Cell cycle assay and flow cytometry

Cells from different groups were incubated for 24 h and isolated by trypsin digestion, washed in pre-chilled PBS with 70% cold ethanol and fixed at 4 °C overnight. The next day, the cells were centrifuged at 1000 g for 5 min and washed with pre-cooled PBS again to harvest the cell pellets. The propidium iodide (PI, 50 μg/mL) staining solution was added to the cell samples and incubated at 37 °C for 30 min in the dark. The cell cycle assay was determined by BD FACSCanto II flow cytometry (BD Biosciences, CA, USA). Cellular DNA content was analyzed using software ModFit LT [32]. The experiment was repeated three times.

### Wounded healing assay

The wounded healing assay was performed as reported previously [33]. In brief, 1 × 10^6^ cells were seeded in a 12-well plate and incubated for 12 h until the cells covered the bottom of the plate. Cells were scribed perpendicularly to the bottom of the plate with a 1 mL gun tip to ensure the same width of each scratch. Cells were washed twice with PBS buffer and cultured with serum-free medium and incubated at 37 °C. Images were acquired every 6 h.

### Human plasma and tissue samples

The human plasma samples were collected from 40 patients with breast cancer and 35 health control checkup population, and 23 pairs of breast cancer and breast cancer adjacent tissues were collected from the First Hospital of Jilin University in 2021. The patients had not received chemotherapy or radiation therapy before surgical resection. The whole blood sample from the K2 EDTA vacuum blood collection tube was centrifuged, the upper plasma sample was drawn and collected in a 1.5 mL Eppendorf tube, and then stored at -80 °C immediately until analysis. The serum and tissue specimens used in the study are the remaining samples after the clinical examination, which were approved by the Human Research Ethics Committee of the First Hospital of Jilin University (Changchun, China). The experiment was performed in accordance with Declaration of Helsinki requirements.

### Immunohistochemistry (IHC) analysis

The human tumor tissues were washed with PBS and fixed in paraformaldehyde for 72 h. After dehydration in ethanol and clearing in xylene, the samples were embedded in paraffin blocks. Paraffin blocks were randomly cut into sections of 4 mm thickness using a tableting machine, incubated at 60 °C for 1 h, dewaxed in xylene, and hydrated by a graded series of ethanol. Endogenous peroxidase was blocked by 3% hydrogen peroxide (H_2_O_2_). The sections were immersed in citrate buffer (pH 6.0) for 5 min under high pressure and cooled naturally. Then the sections were washed with PBS buffer for 10 min and blocked with 5% normal goat serum. Next, the sections were immune-stained with the primary antibodies against LAP3 and ASS1, respectively. After incubation overnight at 4 °C, the samples were washed 3 times (5 min/wash) in PBS, incubated with biotinylated goat anti-multivalent secondary antibody at 37 °C for 30 min and washed another 3 times (5 min/wash) in PBS. Diaminobenzidine (DAB)/H_2_O_2_ reaction was followed by coloration, microscopic observation, and timely termination of tap water washing. After counterstaining with hematoxylin, conventional dehydration, transparentizing, drying, and sealing, the slices were finally photographed under a microscope. This experiment was approved by the Human Research Ethics Committee of the First Hospital of Jilin University (Changchun, China) and performed in accordance with Declaration of Helsinki requirements.

### Statistical analysis

Experimental data were statistically analyzed by GraphPad (version 9.0). Data are presented as the mean ± standard deviation (SD) from three independent replicates. The differences between the mean values of normally distributed data were assessed by one-way ANOVA (Dunnett’s test). *p* < 0.05 was considered statistically significant, which was indicated by "*". *p* < 0.01 indicated that the difference was extremely significant, which was indicated by "**".

## Results

### IFN-γ altered arginine metabolism of BMECs 

As a semi-essential amino acid, arginine metabolism is tightly regulated and comprises a variety of substances [34]. Thus, we speculated that IFN-γ downregulated arginine level possibly by regulating arginine metabolism in BMECs. In order to identify and quantify the arginine metabolites in BMECs upon IFN-γ treatment, we established a targeted metabonomic method for the determination of nine metabolites related to arginine catabolism and anabolism, including arginine, agmatine, creatine, glutamate, putrescine, spermine, ornithine, citrulline and glutamine [35]. Using their corresponding standard solution calibration curves, the concentrations of these compounds were quantified. The chromatographic results showed that all nine metabolite standards and three isotope internal standards (arginine-^13^C6, ornithine-d6 and putrescine-d8) were well separated in their respective channels without interference from impurity peaks (Fig. S1).

**Fig. 1:**
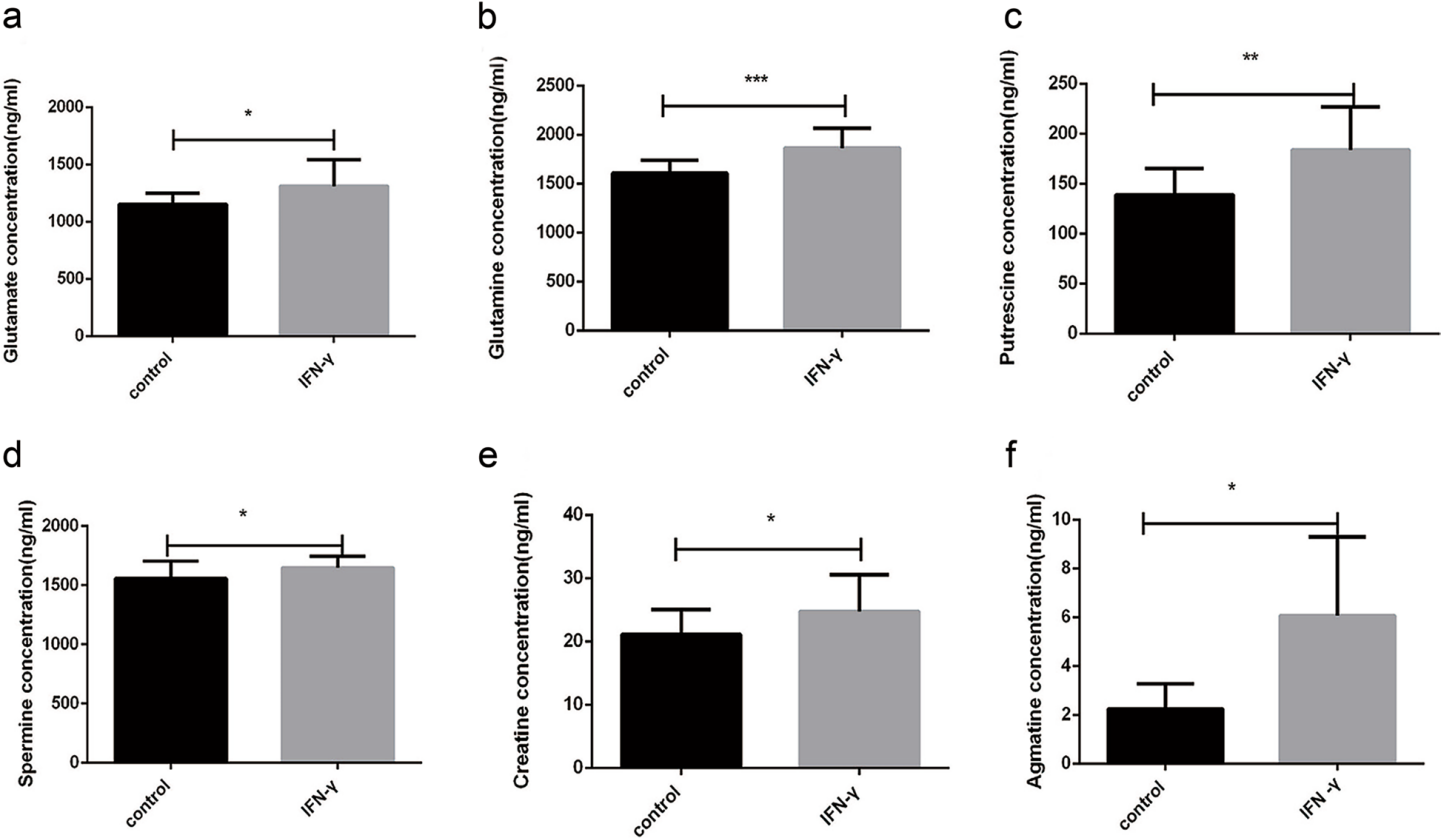
Arginine metabolic alternations of BMECs in response to IFN-γ by targeted metabolomics. BMECs were treatment with 10 ng/mL IFN-γ for 24 h. Targeted metabolomics was used to detect the changes of metabolites related to intracellular arginine metabolic pathway upon IFN-γ treatment. Bars represent mean values with error bars to represent SD from twelve independent replicates. Differences between mean values were assessed by two-tailed Student’s *t*-test. *p < 0.05; **p < 0.01; ***p < 0.001, compared to control

Subsequently, the nine metabolites in BMECs were extracted after treatment with IFN-γ for 24 h and subjected to targeted metabolomics. The results showed that the concentrations of arginine and citrulline, both of which are the major substrates for arginine biosynthesis [36], were both decreased, which was similar to our previous findings [11] (Fig. S2A and B). On the other hand, the concentrations of other metabolites involve in arginine catabolism including glutamate, glutamine, putrescine, spermine, creatine, and agmatine were increased compared with the control group upon IFN-γ treatment (Fig. 1A-F). The concentration of ornithine was not statistically changed (Fig. S2C). Overall, these results suggested that IFN-γ induced arginine depletion through the promotion of arginine catabolism and the inhibition of arginine anabolism in BMECs.

### IFN-γ upregulated LAP3 expression in BMECs

To explore the molecular mechanism for IFN-γ-induced arginine depletion in BMECs, we analyzed the RNA-seq dataset created by our group previously [11]. To validate the RNA-seq data, we selected 19 differentially expressed genes (DEGs) randomly for qRT-PCR analysis and found similar gene expression patterns as the RNA-seq data (Fig. S3A). By comparison of the data of IFN-γ-treated 5th generation of BMECs (N5) to the control (no IFN-γ treatment, NC), a total of 3146 DEGs were discovered (2528 genes upregulated and 618 genes downregulated), with false discovery rate (FDR) ≤ 0.01 and |log2 Ratio|≥ 1 (Fig. S3B). To characterize the functional changes of BMECs in response to IFN-γ, we performed KEGG pathway and GO enrichment analysis with DAVID 6.8 [37]. We found that the majority of the DEGs were associated with cell growth pathways including cell cycle, inflammation pathways including mTOR and MAPK signaling, tumor pathways including melanoma and nonsmall cell lung cancer (Fig. S4A and B).

**Fig. 2:**
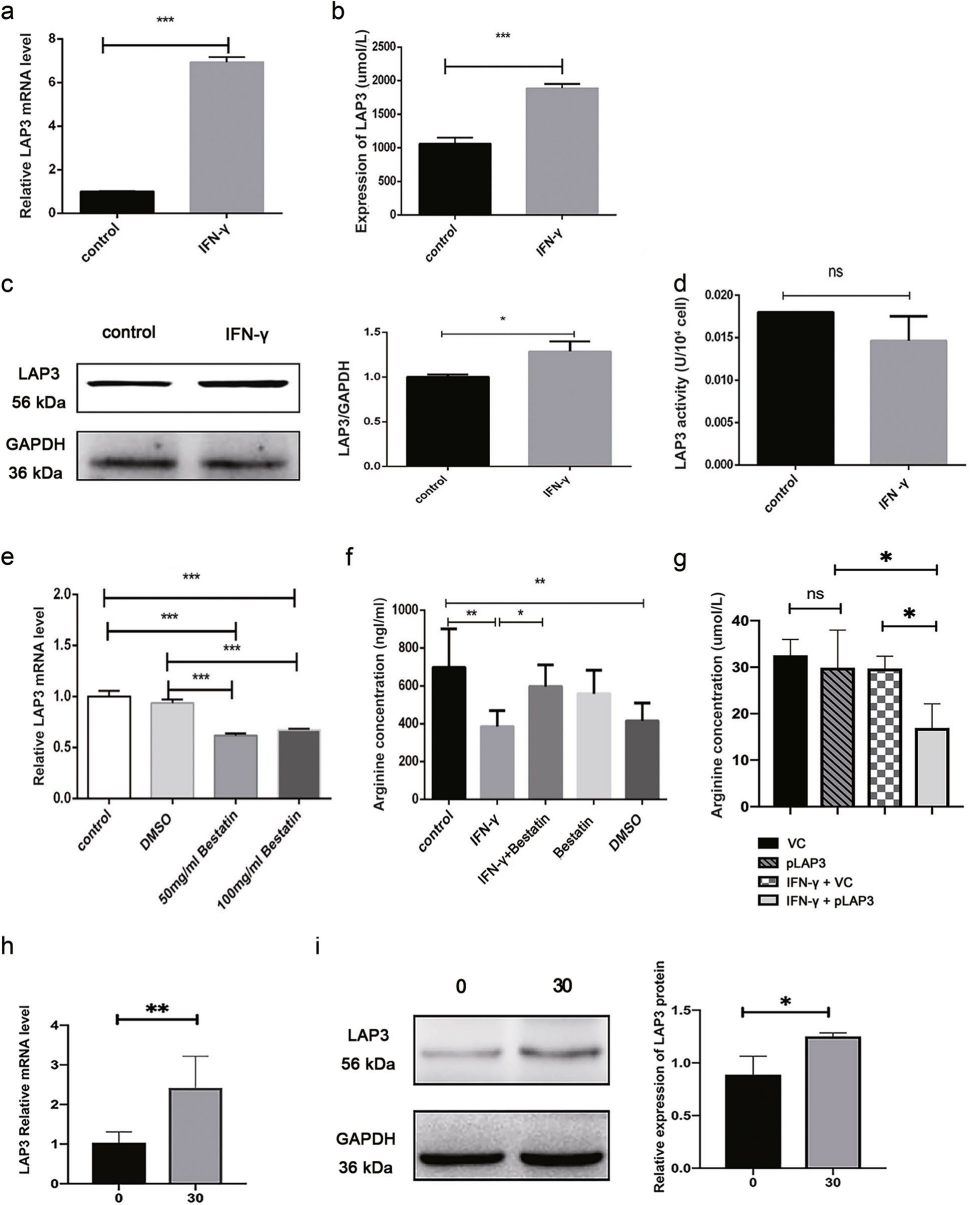
IFN-γ upregulated LAP3 expression in BMECs. **a** qRT-PCR was used to detect the transcription level of *LAP3* in BMECs after treatment with IFN-γ. **b** ELISA confirmed the content of LAP3 after treatment with IFN-γ in BMECs. **c** Western blotting was used to test the expression level of LAP3 in BMECs after treatment with 10 ng/mL IFN-γ for 24 h. Full-length blots are presented in Supplementary Fig. S5. **d** Leucine aminopeptidase activity was used to detect the change of LAP3 enzyme activity in BMECs before and after IFN-γ treatment. **e** Analysis of intracellular LAP3 expression in BMECs treated with the LAP3 inhibitor, bestatin, for 12 h using qRT-PCR. **f** HPLC was used to detect the changes of arginine content in BMECs pretreated with 100 mg/mL bestatin for 12 h and stimulated by IFN-γ for 24 h. **g** Changes in arginine content upon LAP3 overexpression in BMECs with or without IFN-γ treatment, as determined by ELISA. VC, pLenti-CMV-GFP-Puro. pLAP3, pLenti-CMV-LAP3-GFP-Puro. **h-i** LAP3 expression in normal and malignant BMECs by qRT-PCR (**h**) and western blotting analysis (**i**). 0, no IFN-γ treatment, normal BMECs; 30, malignant BMECs after IFN-γ-treated for 30th generations. The relative level of the target protein was estimated using densitometry and the ratio was calculated relative to the GAPDH control. Full-length blots are presented in Supplementary Fig. S6. Bars represent mean values with error bars to represent SD from three independent replicates. Differences between mean values were assessed by two-tailed Student’s *t*-test. *p < 0.05; **p < 0.01; ***p < 0.001, compared to control

In order to identify novel genes and pathways, we focused on the genes that have not been described as modifying arginine metabolism in the literatures. Ranking these genes by *p* value, we found that that *LAP3* was one of the most significantly changed genes in response to IFN-γ treatment (Fig. S3B). We then verified this result by western blotting, qRT-PCR and ELISA analysis. The results showed that both the mRNA and protein expression levels of LAP3 was robustly upregulated upon IFN-γ treatment compared with the control group in BMECs (Figs. 2A-C). The enzymatic activity of LAP3 was not affected upon treatment with IFN-γ (Fig. 2D).

To determine whether LAP3 involves in IFN-γ-induced arginine depletion, the expression of LAP3 in BMECs was inhibited using LAP3 small-molecule protein inhibitor, bestatin (Fig. 2E), and the arginine concentration upon IFN-γ coincubation was detected by high performance liquid chromatography-mass spectrometry (HPLC–MS/MS). The results showed that bestatin pretreatment significantly alleviated the IFN-γ-induced downregulation of arginine level in BMECs (Fig. 2F). Consistently, overexpression of LAP3 using a plasmid decreased the arginine level in BMECs upon IFN-γ induction (Fig. 2G). Besides arginine depletion, long-term treatment with IFN-γ also induces the malignant transformation of BMECs [17, 33]. Notably, the mRNA and protein expression level of LAP3 was also upregulated in malignant BMECs compared to the normal BMECs, as detected by qRT-PCR and western blotting (Fig. 2H and I), indicating a critical role of LAP3 in the regulation of IFN-γ-induced malignant transformation of BMECs. Together, these results suggested that IFN-γ induced arginine depletion through upregulation of LAP3 in BMECs.

### LAP3 downregulated arginine level by interfering with ASS1

**Fig. 3:**
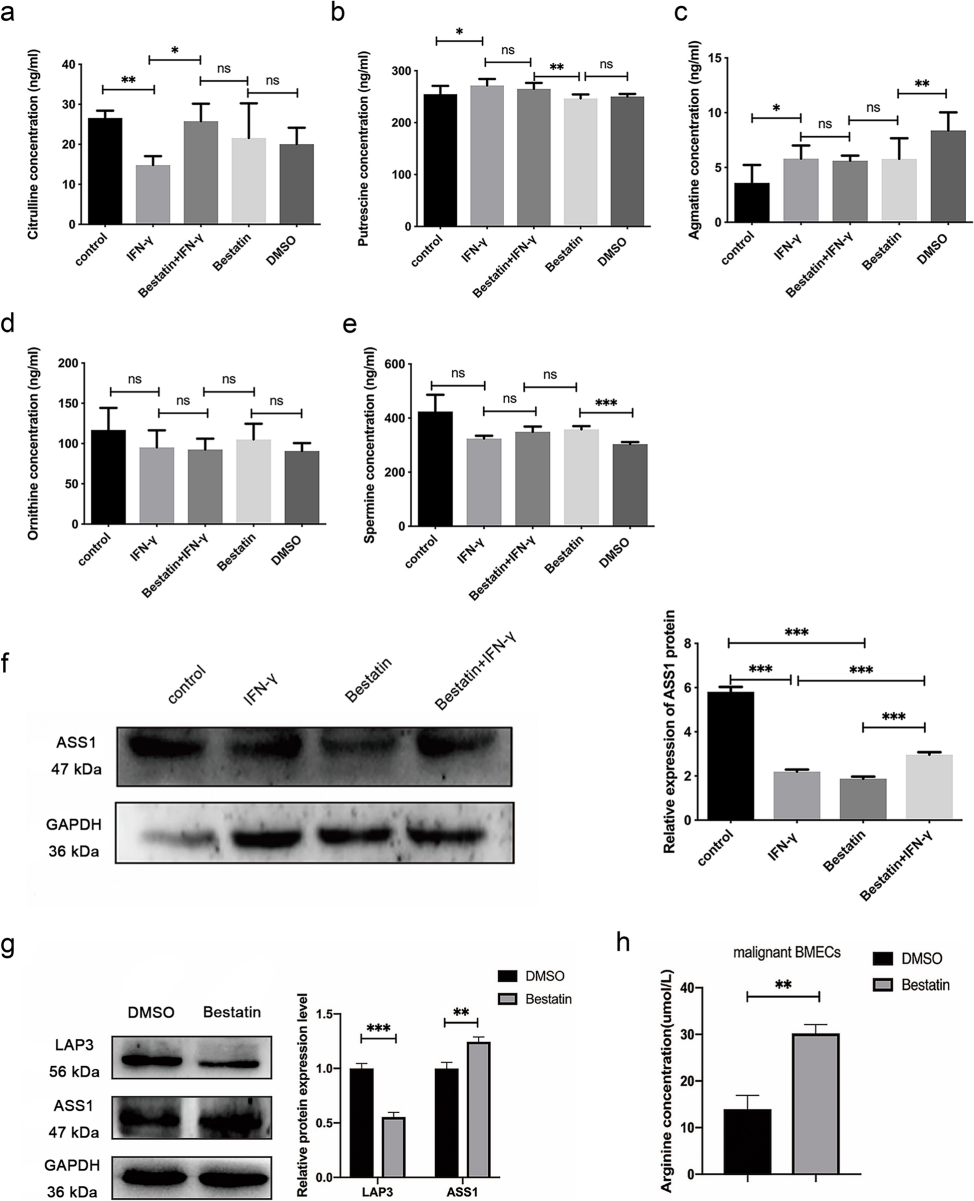
LAP3 downregulates arginine level by interfering with ASS1 in BMECs. **a-e** Targeted metabolomics were performed to detect intracellular levels of citrulline (**a**), putrescine (**b**), agmatine (**c**), ornithine (**d**), and spermine (**e**) with IFN-γ or in combination with 100 mg/mL LAP3 inhibitor, bestatin. **f** Western blotting was used to detect the expression of ASS1 protein in LAP3 knocked-down BMECs upon IFN-γ treatment. Full-length blots are presented in Supplementary Fig. S7. **g** Western blotting showed the changes of ASS1 protein expression after treatment with LAP3 inhibitor bestatin in malignant transformed BMECs cells. Full-length blots are presented in Supplementary Fig. S8. **h** Determination of the change in arginine content of malignant BMECs treated with the LAP3 inhibitor, bestatin. The relative level of the target protein was estimated using densitometry and the ratio was calculated relative to the GAPDH control. Bars represent mean values with error bars to represent SD from three independent replicates. Differences between mean values were assessed by two-tailed Student’s *t*-test. *p < 0.05; **p < 0.01; ***p < 0.001, compared to control

Subsequently, we examined the levels of arginine metabolites upon treatment of IFN-γ and bestatin through targeted metabolomics (Fig. 3A-E). Citrulline is the major substrate for arginine biosynthesis [36]. The results showed that inhibition of LAP3 by its inhibitor bestatin also significantly alleviated the IFN-γ-induced downregulation of citrulline in BMECs (Fig. 3A), suggesting a role of LAP3 in inhibition of arginine biosynthesis in BMECs upon IFN-γ treatment. On the other hand, coincubation of IFN-γ with its inhibitor bestatin did not change the IFN-γ-induced upregulation of putrescine and agmatine levels (Fig. 3B and C), both of which are products of arginine catabolism, suggesting LAP3 did not regulate arginine catabolism in BMECs upon IFN-γ treatment. The concentration of ornithine and spermine was not statistically changed in BMECs upon IFN-γ treatment alone or in combination with bestatin (Fig. 3D and E). These results suggested that LAP3 contributed to IFN-γ-induced arginine depletion through inhibition of arginine biosynthesis, but not arginine catabolism in BMECs.

ASS1 is a key rate-limiting enzyme in the arginine biosynthesis pathway [13]. To further investigate the mechanism of LAP3 on arginine biosynthesis, the expression of ASS1 was assessed. Western blotting analysis showed that ASS1 expression was dramatically downregulated upon IFN-γ treatment, which was in consistent with previous findings [11]. However, coincubation of IFN-γ with LAP3 inhibitor bestatin significantly alleviated the IFN-γ-induced inhibition of ASS1 expression (Fig. 3F). Similarly, treatment with LAP3 inhibitor bestatin downregulated LAP3 expression, but upregulated the ASS1 expression in malignant BMECs (Fig. 3G). As expected, the arginine level was significantly increased when treated the malignant BMECs with the LAP3 inhibitor bestatin (Fig. 3H). Overall, these results indicated that LAP3 involved in the arginine metabolism via interfering with ASS1 in both normal and malignant BMECs.

### LAP3 promoted cell cycle of BMECs 

**Fig. 4:**
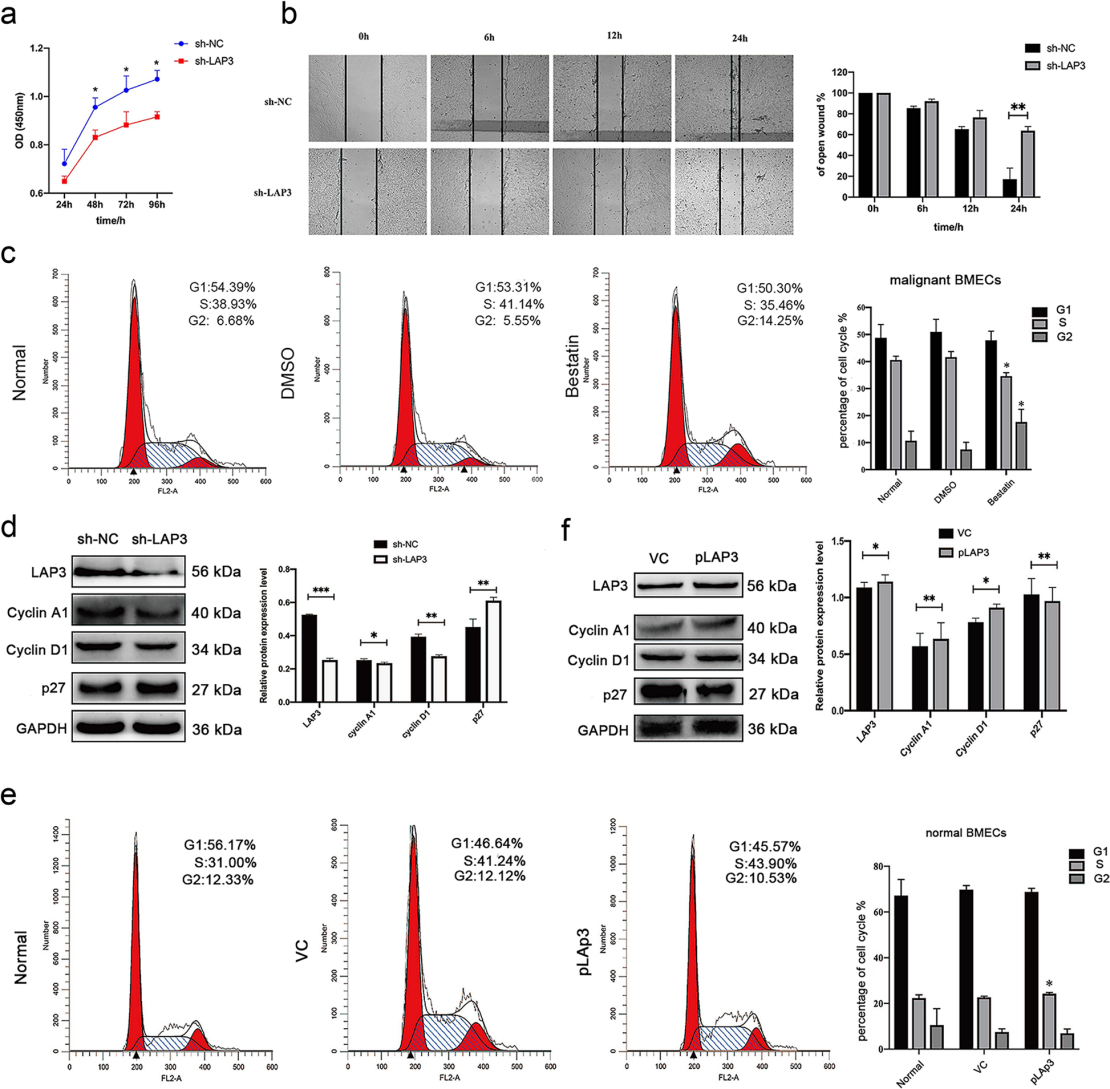
Upregulation of LAP3 promoted cell cycle process of BMECs. **a** Detection of cell proliferation using CCK-8 method. **b** Effect of LAP3 on cell migration by cell scratch test. **c** LAP3 affects the cell cycle distribution of malignant transformed BMECs. The malignant transformed BMECs were treated with the LAP3 inhibitor bestatin, which was stained with propidium iodide (PI) and detected for DNA content and cell cycle distribution by flow cytometry. **d** Western blotting analyzed the effect of knock-down of *LAP3* on cyclin A1, cyclin D1, and p27 expressions in malignant BMECs. Full-length blots are presented in Supplementary Fig. S9. **e** Overexpression of LAP3 promoted the cell cycle of normal BMECs. LAP3 was overexpressed using a plasmid in normal BMECs. Cells were stained with propidium iodide (PI) and detected for DNA content and cell cycle distribution by flow cytometry. **f** Western blotting analyzed the effect of overexpression of LAP3 on cyclin A1, cyclin D1, and p27 expressions in normal BMECs. VC, pLenti-CMV-GFP-Puro. pLAP3, pLenti-CMV-LAP3-GFP-Puro. Full-length blots are presented in Supplementary Fig. S10. The relative level of the target protein was estimated using densitometry and the ratio was calculated relative to the GAPDH control. Bars represent mean values with error bars to represent SD from three independent replicates. Differences between mean values were assessed by two-tailed Student’s *t*-test. *p < 0.05; **p < 0.01; ***p < 0.001, compared to control

LAP3 has been shown to involve in malignant development of human breast cancer cells [25], thus we wonder if LAP3 also participates in the malignant transformation of BMECs. The cell proliferation and wound healing assays were performed, and the expression of LAP3 was knocked-down using a shRNA interfering plasmid (sh-LAP3). We found that the cell growth rates of *LAP3* knocked-down cells were significantly lowered compared with control cells (Fig. 4A), indicating LAP3 promoted the cell proliferation of malignant BMECs. In addition, wound healing experiments showed that knocked-down of *LAP3* displayed a slower cell migration than that of control cells, indicating LAP3 also promoted cell migration (Fig. 4B). To further investigate the effects of LAP3 on cell proliferation, we applied flow cytometry and western blotting to detect the cell cycle phase transition and the expression of cell cycle proteins, respectively. Treatment of malignant BMECs with LAP3 inhibitor bestatin significantly reduced the percentage of cells in S phase, but increased in G2 phase compared with control group (Fig. 4C), indicating LAP3 promoted S/G2 transition of cell cycle. Detection of the key cell cycle regulatory proteins revealed that knocked-down of *LAP3* downregulated the expression of cyclin A1 and cyclin D1, but upregulated the expression of the cell cycle negative regulator p27 in malignant BMECs (Fig. 4D). To further confirm these results, we overexpressed LAP3 on a plasmid in normal BMECs. Elevated levels of LAP3 increased the percentage of cells in S phase, but decreased the percentage of cells in G2 phase (Fig. 4E). Moreover, LAP3 overexpression also upregulated the expression of cyclin A1 and cyclin D1, but downregulated the expression of cell cycle negative regulator p27 in normal BMECs (Fig. 4F). These results suggested that LAP3 contributed to malignant transformation of BMECs by promotion of cell cycle process.

### LAP3 promoted cell cycle of malignant BMECs by upregulation of HDAC2

**Fig. 5:**
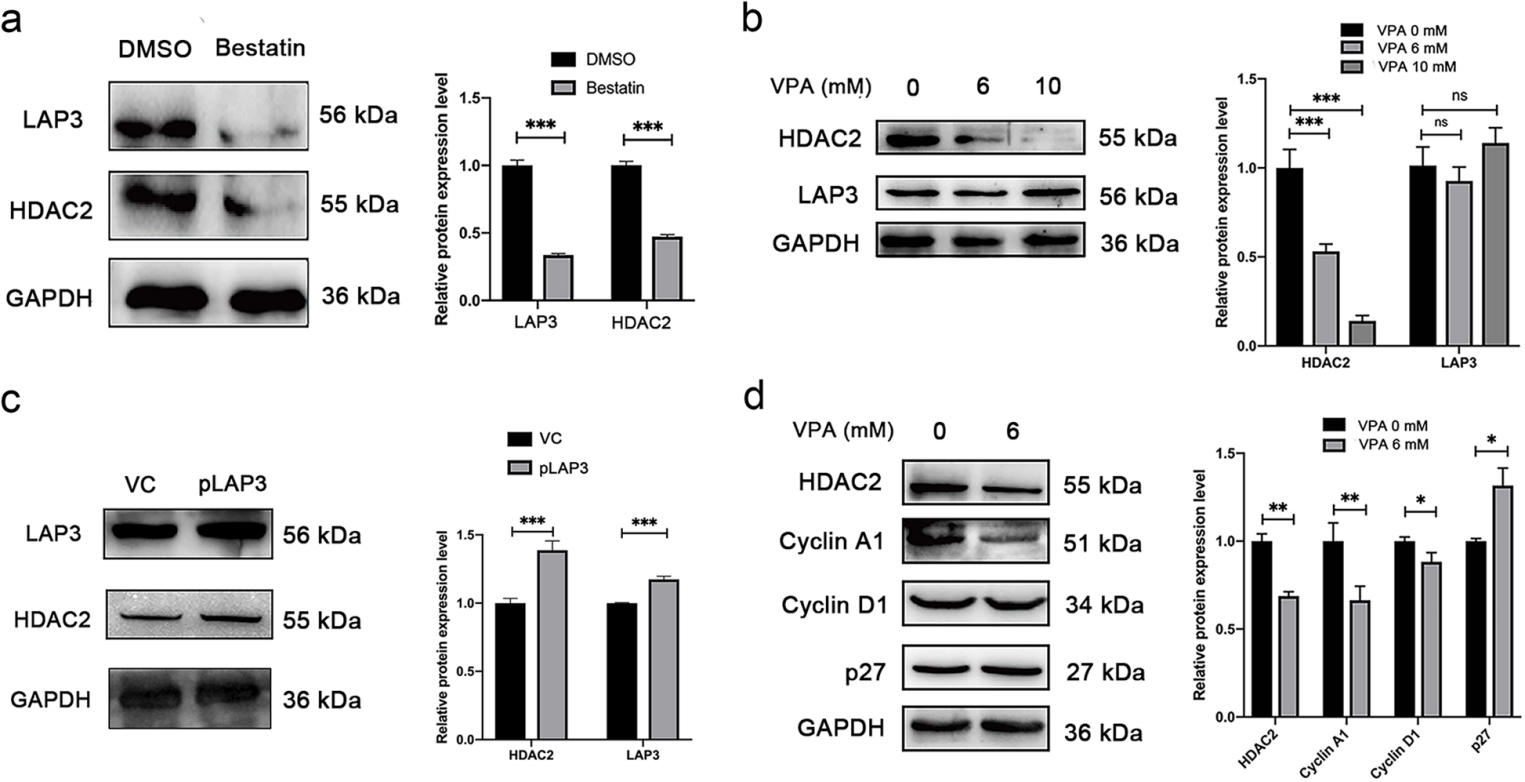
Knock-down of HDAC2 abolished the acceleration of cell cycle induced by LAP3 upregulation in BMECs. **a** Effect of LAP3 inhibitor bestatin on LAP3 and HDAC2 protein expressions in malignant BMECs. Full-length blots are presented in Supplementary Fig. S11. **b** Effect of HDAC2 inhibitor VPA on HDAC2 and LAP3 protein expressions in malignant BMECs. Full-length blots are presented in Supplementary Fig. S12. **c** Effect of LAP3 overexpression on LAP3 and HDAC2 protein expressions in normal BMECs. VC, pLenti-CMV-GFP-Puro. pLAP3, pLenti-CMV-LAP3-GFP-Puro. Full-length blots are presented in Supplementary Fig. S13. **d** Effect of HDAC2 inhibitor VPA on cell cycle proteins cyclin A1, cyclin D1, and p27 expressions in malignant BMECs. Full-length blots are presented in Supplementary Fig. S14. The relative level of the target protein was estimated using densitometry and the ratio was calculated relative to the GAPDH control. Bars represent mean values with error bars to represent SD from three independent replicates. Differences between mean values were assessed by two-tailed Student’s *t*-test. *p < 0.05; **p < 0.01; ***p < 0.001, compared to control

HDAC2 is a histone deacetylase that has been shown to regulate IFN-γ-induced malignant cell growth [38]. Therefore, we speculated that LAP3 may be involved in the process of malignant transformation of BMECs by regulation of HDAC2. As expected, treatment with LAP3 inhibitor bestatin downregulated the HDAC2 expression, while inhibition of HDAC2 expression by its inhibitor valproic acid (VPA) did not changed the expression of LAP3 in malignant BMECs (Fig. 5A and B). In normal BMECs, the HDAC2 expression was also upregulated upon overexpression of LAP3 on a plasmid (Fig. 5C), which indicated that HDAC2 was positively regulated by LAP3 in BMECs. Subsequently, we detected the expression levels of cell cycle regulators upon inhibition of HDAC2 expression in malignant BMECs to investigate the role of HDAC2 in IFN-γ-induced malignant transformation. The results demonstrated that inhibition of HDAC2 expression by VPA downregulated the expression of cyclin A1, cyclin D1, but upregulated the expression of p27 in malignant BMECs (Fig. 5D). Together, these results indicated that LAP3 promoted cell cycle process of malignant BMECs by upregulation of HDAC2.

### Arginine supplementation did not affect LAP3 expression, but slowed down cell cycle of malignant BMECs

**Fig. 6:**
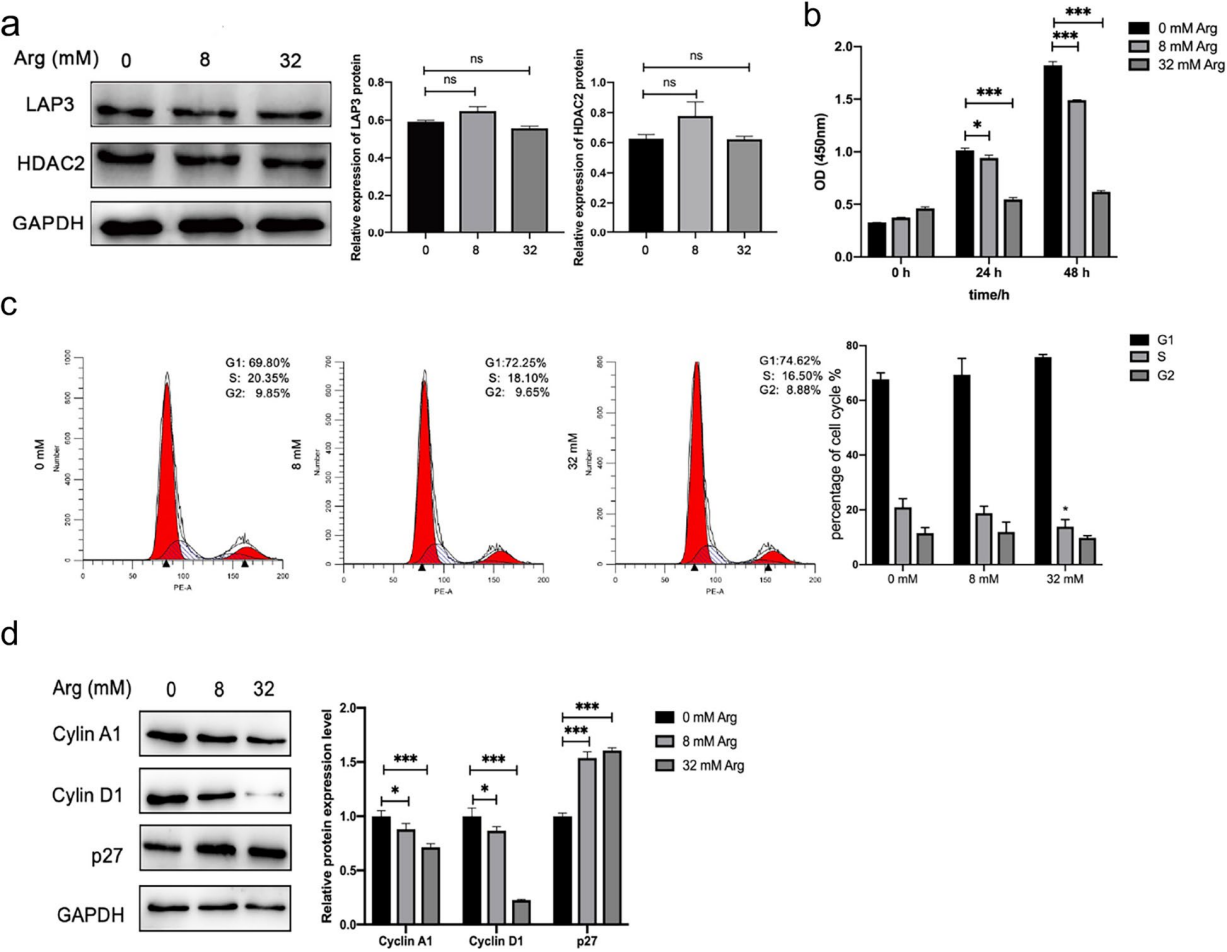
Arginine antagonized cell cycle of malignant BMECs. **a** Detection of LAP3 and HDAC2 expressions in malignant BMECs supplemented with arginine. **b** Detection of cell proliferation using CCK-8 method. **c** Arginine supplementation affected the cell cycle distribution of malignant BMECs. Malignant BMECs were treated with 8 mM and 32 mM arginine for 48 h, followed by staining with propidium iodide (PI) for DNA content and cell cycle distribution by flow cytometry. **d** Western blotting analyzed the effect of arginine supplementation on cyclin A1, cyclin D1, and p27 expressions in malignant BMECs. Malignant BMECs were treated with 8 mM and 32 mM arginine for 48 h, and the expressions of LAP3, HDAC2, cyclin A1, cyclin D1, and p27 were detected by western blotting. Full-length blots are presented in Supplementary Fig. S11 and S15, respectively. The relative level of the target protein was estimated using densitometry and the ratio was calculated relative to the GAPDH control. Bars represent mean values with error bars to represent SD from three independent replicates. Differences between mean values were assessed by two-tailed Student’s *t*-test. *p < 0.05; **p < 0.01; ***p < 0.001, compared to control

Previously we have shown that arginine supplementation antagonized IFN-γ-induced malignant transformation of BMECs [33], thus we wonder if arginine supplementation also affects LAP3 expression. Interestingly, addition of arginine up to 32 mM did not alter the LAP3 expression, nor the HDAC2 expression in malignant BMECs after 48 h incubation (Fig. 6A). On the other hand, arginine supplementation inhibited cell proliferation of malignant BMECs, as demonstrated by CCK-8 analysis (Fig. 6B). Furthermore, detection of cell cycle phase transition by flow cytometry showed that arginine supplementation slightly increased the percentage of cells in G1 phase, but significantly reduced the percentage of cells in S phase (Fig. 6C), indicating arginine downregulated G1/S transition of cell cycle. Accordingly, both the expression of the cell cycle protein cyclin A1 and cyclin D1 was downregulated along with arginine supplementation in malignant BMECs, while the expression of p27 was upregulated (Fig. 6D). Overall, these results indicated that arginine antagonized malignant transformation of BMECs through interfering with cell cycle process.

### IFN-γ upregulated LAP3 expression by regulation of p38 and ERK MAPKs in BMECs 

**Fig. 7:**
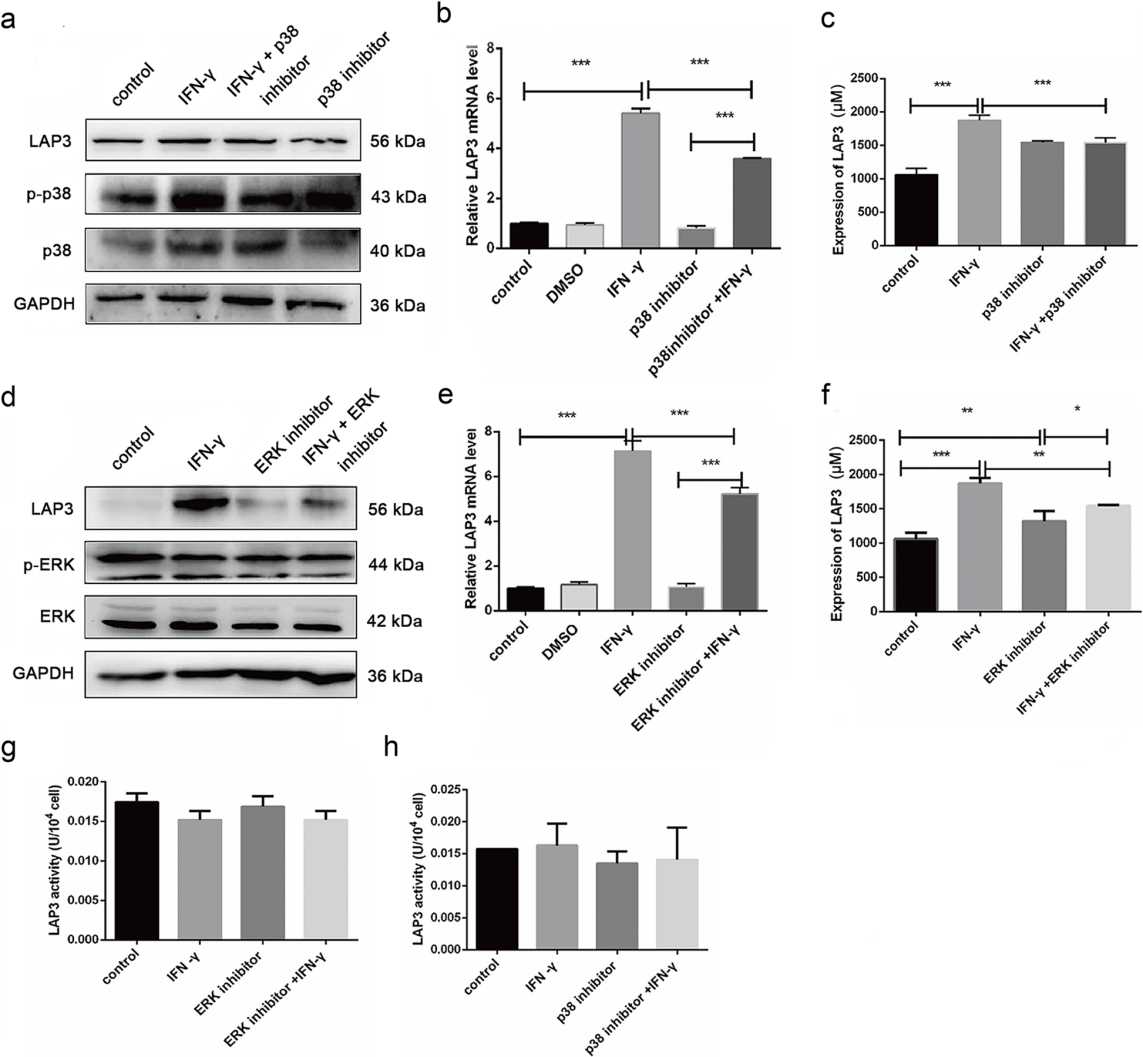
IFN-γ upregulated LAP3 by regulation of p38 and ERK MAPKs in BMECs. **a-h** BMECs were treated with either IFN-γ alone or co-incubated with p38 and ERK inhibitor for 24 h. The expression (**a-f**) and enzymatic activity (**g, h**) of LAP3 was detected by western blotting (**a, d**), qRT-PCR (**b, e**), and ELISA (**c, f-h**), respectively. For western blotting, the relative level of the target protein was estimated using densitometry and the ratio was calculated relative to the GAPDH control. Full-length blots are presented in Supplementary Fig. S16 (**a**) and S17 (**d**). Bars represent mean values with error bars to represent SD from three independent replicates. Differences between mean values were assessed by two-tailed Student’s *t*-test. *p < 0.05; **p < 0.01; ***p < 0.001, compared to control

Bioinformatic analysis of the transcriptome upon IFN-γ treatment indicated that MAPK signaling involved in malignant transformation of BMECs (Fig. S4B). As expected, IFN-γ treatment upregulated the expression of MAPK family member protein p38 and p-p38 along with upregulated LAP3 expression (Fig. 7A-C), indicating p38 MAPK signaling pathway was activated upon IFN-γ induction in BMECs. On the other hand, IFN-γ treatment downregulated the expression of p-ERK, but did not affected the expression of ERK upon IFN-γ induction in BMECs (Fig. 7D). To further assess if p38 and ERK MAPKs regulate LAP3, we blocked the expression of p38 and ERK using respective protein inhibitors and then assessed the LAP3 expression either with or without IFN-γ. The results showed that either block of p38 and ERK significantly reduced the IFN-γ-induced upregulation of LAP3 both at the transcription and protein levels, regardless of with or without IFN-γ treatment in BMECs (Fig. 7A-F). In addition, treatment with ERK and p38 MAPK inhibitors either alone or in combination with IFN-γ did not affect LAP3 enzyme activity in BMECs (Fig. 7G and H). Overall, these results indicated that IFN-γ upregulated LAP3 by activation of p38 MAPK and inhibition of ERK MAPK signaling in BMECs.

### LAP3 was highly expressed in human breast cancer tissues

**Fig. 8:**
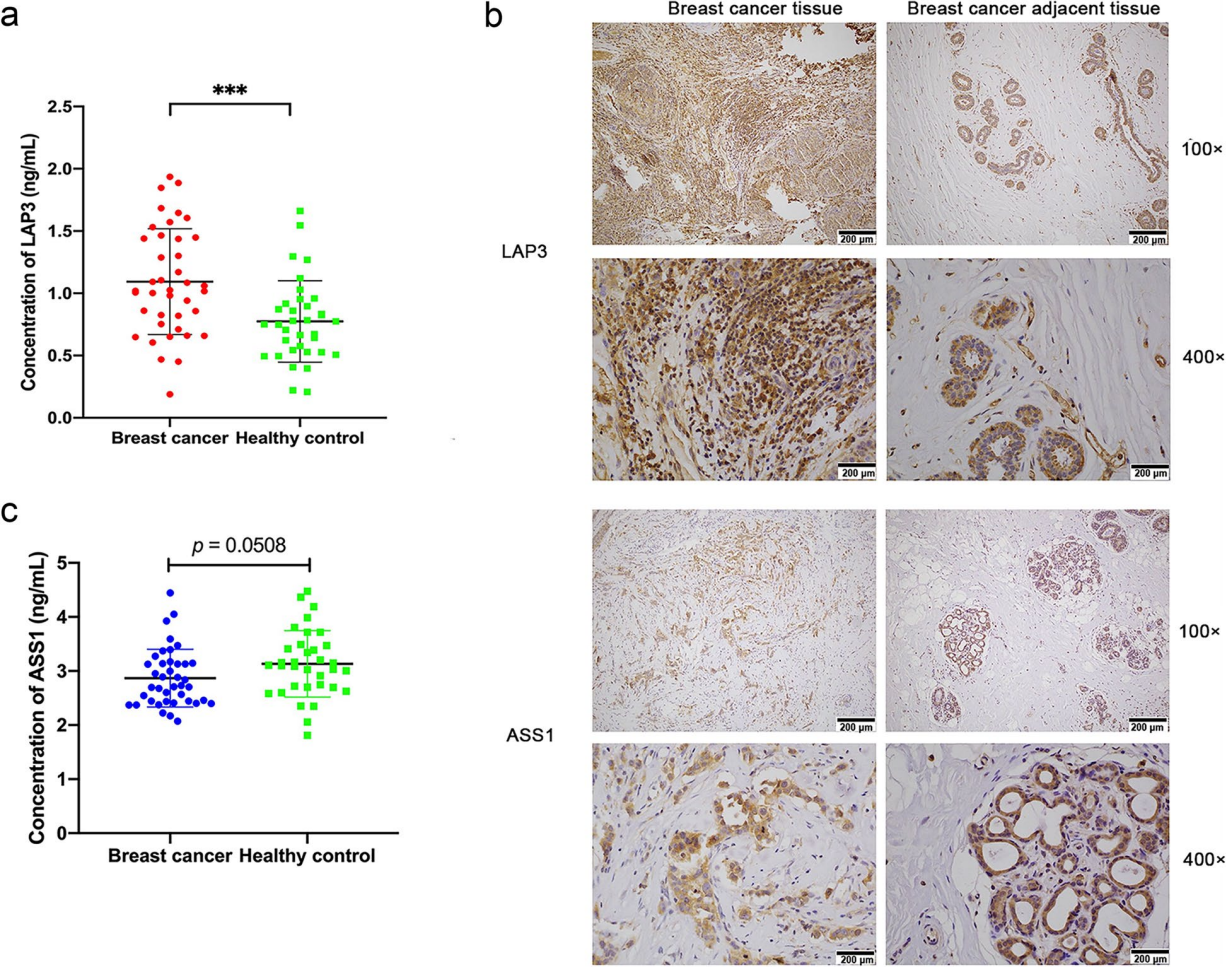
LAP3 was highly expressed in human breast cancer samples. **a, c** ELISA experiment was used to detected the protein expression levels of LAP3 (**a**) and ASS1 (**c**) in plasma of breast cancer patients and healthy control. Bars represent mean values with error bars to represent SD from three independent replicates. Differences between mean values were assessed by two-tailed Student’s *t*-test. *p < 0.05; **p < 0.01; ***p < 0.001, compared to control. **b** Representative images of IHC staining of LAP3 and ASS1. Scale bar, 200 μm

To better understand the effect of LAP3 on tumorigenesis, human serum and tissue samples from breast cancer patients were collected in Department of Clinical Laboratory and Department of Pathology, the First Hospital of Jilin University. The expression levels of LAP3 in the serum of 40 breast cancer patients and 35 healthy people were detected by ELISA, and the results showed that the average concentration of LAP3 in the serum of breast cancer patients was 1.09 ± 0.43 ng/mL, which was higher than that in the serum of healthy control population (0.77 ± 0.33 ng/mL). The difference between the concentration of LAP3 in the serum of the two groups was significant (*p* < 0.001) (Fig. 8A). In addition, the immunohistochemical (IHC) showed that positive expression of LAP3 in the breast cancer tissues increased dramatically compared with that in the adjacent tissues (Fig. 8B).

Previously we demonstrated that the arginine level in both the human plasma and the tissue specimens of breast cancer patients was significantly lower than that in healthy human plasma and the tissue specimens of the adjacent tissues [33]. As the key enzyme for arginine biosynthesis, we speculated that the expression of ASS1 in patients with breast cancer might also be different compared with that in normal people. The results showed that the average concentration of ASS1 in serum of patients with breast cancer was 2.868 ± 0.53 ng/mL, which was lower than that in the serum of healthy control population (3.133 ± 0.615 ng/mL, *p* = 0.0508) (Fig. 8C). Lower expression of ASS1 was also observed in breast cancer tissues than the adjacent tissues (Fig. 8B). Together, these results supported that LAP3 contributed to tumorigenesis and malignancy of human breast.

## Discussion

Previously we demonstrated that sustained IFN-γ led to arginine depletion in bovine mammary gland and contributed to malignant transformation of BMECs [2, 9, 17]. In this work, we showed that IFN-γ induced arginine depletion by promotion of arginine catabolism and inhibition of arginine anabolism in BMECs. We identified that LAP3, which interferes with the expression of arginine synthase ASS1, mediated IFN-γ-induced arginine depletion via p38/ERK MAPKs signaling pathways. LAP3 also contributed to the malignant transformation through accelerating cell cycle of BMECs. In human breast cancer tissues, LAP3 was highly expressed. These findings unveiled the molecular mechanism for IFN-γ-induced arginine depletion and malignant transformation of BMECs, and highlighted the role of LAP3 as a potential therapeutic target for arginine related disease both in human and dairy cows.

IFN-γ is a Th1 (T helper cell type 1) type pleiotropic cytokine that affects both host defense and immune regulation [39]. Unlike TNF-α, which causes acute inflammation upon release, IFN-γ functions in chronic infection through modulation of the release of other inflammatory mediators, such as TNF-α, reactive oxygen species, and nitric oxide, and contributes to carcinogenesis [40]. As a proinflammatory cytokine, short-term stimulation by IFN-γ facilitates the adhesion and invasion of *S. aureus*, a frequent pathogen in bovine mammary gland [41], into BMECs and leads to mastitis [2, 11]. Additionally, short-term stimulation of IFN-γ can activate B-catenin which promotes cell proliferation to regulate epithelial cell homeostasis [42]. Inflammation is a critical component of tumor progression, it can initiate or exacerbate carcinogenesis [40, 43]. Although numerous studies have shown that IFN-γ is vital to tumor surveillance by the immune system, it also has protumorigenic effects under certain circumstances [14, 44, 45]. For example, sustained low-level of IFN-γ exposure promotes the development of MA782/5S mammary adenocarcinoma [15, 16]. Consistently, our previous findings demonstrated that corn straw-based cow diets induced increased levels of IFN-γ, and long-term stimulation by IFN-γ induced malignant transformation of BMECs [9, 17], a precancerous phenotype with drastic cell morphology and function alternations. These studies indicate a dual role of IFN-γ in regulation of bovine mammary gland inflammation and carcinogenesis. In this study, we identified and demonstrated that LAP3 contributed to IFN-γ-induced malignant transformation of BMECs.

Arginine is an important metabolite for carcinogenesis. Tumors requires large amounts of arginine for cellular functions. However, many tumor types, such as melanoma, hepatocellular carcinoma, and pancreatic cancer, are arginine auxotrophic because of silencing in ASS1 expression, which means the cancer cells are devoid of endogenous arginine and therefore have a critical dependence on exogenous arginine [35, 46–48]. Although arginine deficiency causes immunosuppression, arginine auxotrophs could be exploited to treat many cancers, including breast cancers. On the other hand, arginine can be produced from citrulline in non-auxotrophic tumors and studies have shown that arginine supplementation improves the results of treatment of patients with arginine non-auxotrophic cancer, such as head and neck squamous cell carcinoma [49]. Intratumoral injection of the arginine-producing bacteria in combination anti-PD-L1 treatment improved tumor clearance and T cell memory [50]. In this study, we found that arginine supplementation could inhibit malignant transformation of BMECs by slowing down cell cycle process, indicating arginine antagonized the IFN-γ-induced cell malignant transformation.

Arginine is a conditionally essential amino acid with multiple biological functions both in humans and animals. Arginine metabolism is tightly regulated by diet, cytokines and hormone, and thus participate in occurrence of various diseases. Tang et al. assessed arginine with its catabolic products and showed that diminished arginine and high citrulline levels are associated with the incidence of cardiovascular disease [51]. Arginine depletion reduces the secretion of cytokines by immune cells and increases susceptibility to *S. aureus*, a frequent pathogen for bovine mastitis [11]. Moreover, sustained exposure to IFN-γ results in arginine depletion which leads to substantial alternations in normal functionality of bovine mammary gland including malignant transformation and impaired milk protein and fat synthesis of BMECs[2, 17, 33]. Consistently, KEGG pathway and GO enrichment analysis of the DEGs upon IFN-γ induction demonstrated the majority of them were associated with cell growth pathways including cell cycle, inflammation pathways including mTOR and MAPK signaling, tumor pathways including melanoma and nonsmall cell lung cancer (Fig. S4A and B).

Besides dietary supply, arginine homeostasis largely depends on endogenous synthesis, catabolism, and arginine transportation through cell membranes, which are directly or indirectly regulated by large numbers of proteins [52]. In combination with previous findings [11], we showed that IFN-γ induced arginine depletion by promotion of arginine catabolism and the inhibition of arginine anabolism in BMECs (Fig. 1; Fig. S2). Furthermore, analysis of the transcriptome upon IFN-γ stimulation identified LAP3, which downregulated arginine level by interfering with ASS1, a key rate-limiting enzyme in arginine biosynthesis of BMECs. Besides *LAP3*, many other DEGs were also identified upon IFN-γ stimulation in BMECs. However, whether theses DEGs also involve in IFN-γ-induced arginine depletion needs further investigated.

Leucine aminopeptidase (LAPs) family proteins play vital roles in protein quality control and cell proliferation and differentiation [53]. Dysregulation of LAPs is associated with many diseases including liver dysfunction, pancreatic and bile duct diseases, angiogenesis, and carcinogenesis [19, 54]. LAP3 is one member of the LAPs and has been reported to be upregulated in diverse cancer types, such as ovarian cancer, glioma, esophageal squamous cell carcinoma and hepatocellular carcinoma [21–25]. However, the detailed mechanism of LAP3 on cell malignant transformation has not been reported. In this study, we found that LAP3 not only promoted cell cycle process by regulating HDAC2 expression, but also involved in arginine depletion through inhibition of ASS1 which leads to malignant transformation of BMECs upon IFN-γ stimulation in BMECs.

HDAC2 is a Rpd3-like class I protein that belongs to the histone deacetylases family [55]. HDAC2 is highly expressed in human tumor tissues, and overexpression of HDAC2 induces tumor cell proliferation, blocks apoptosis and promotes tumorigenesis [56]. HDAC2 also affects the proliferation of gastric cancer cells by regulating cell cyclins and plays a role in Myc-mediated tumorigenesis [57]. Similarly, our previous study found that the IFN-γ-induced malignant growth of BMECs is regulated by c-Abl/HDAC2 signaling pathway [38]. Acetylation and deacetylation of histone proteins play an important role in chromatin remodeling and transcriptional regulation. HDACs catalyzes the removal of acetyl groups to specific lysine-rich amino terminal histone residues [58]. We found that knock-down of HDAC2 downregulated the expression of cyclin A1, cyclin D1, but upregulated the expression of p27 in malignant BMECs. However, whether the observed phenomenon is directly or indirectly regulated by HDAC2 catalytic activity still needs to be confirmed.

In summary, this study demonstrated that sustained IFN-γ led to arginine depletion and malignant transformation by upregulation of LAP3 in BMECs. These results indicate a close relationship between cytokines, metabolism and tumorigenesis of mammary gland. It also provides a novel therapeutic target for breast cancer both in dairy cows and human.

## Supplementary Information

**Additional file 1: Figure S1**. Targeted metabolomics for internal standards. All the internal standards were well separated without interfering peaks.** Figure S2**. The levels of arginine, ornithine, citrulline in BMECs upon IFN-γ treatment using targeted metabolomics. BMECs were treatment with 10 ng/mL IFN-γ for 24 h. Targeted metabolomics was used to detect the changes of arginine (a), citrulline (b), and ornithine (c) upon IFN-γ treatment. Bars represent mean values with error bars to represent SD from twelve independent replicates. Differences between mean values were assessed by two-tailed Student’s t-test. *p < 0.05; **p < 0.01; ***p < 0.001, compared to control.** Figure S3**. qRT-PCR validation of RNA-seq data (a) and volcano plots for the DEGs (b). (a) 19 DEGs from the RNA-seq data were selected for qRT-PCR analysis. (b) Left, a volcano plot showing RNA-seq results of IFN-γ-treated 5th generation of BMECs (N05) to the control (no IFN-γ treatment, NC). Downregulated or upregulated genes were divided by |log2Ratio| ≥1 with false discovery rate (FDR) ≤ 0.01. Red dots for upregulated genes and blue dots for downregulated genes. Right, depiction of selected 10 most significantly changed genes.** Figure S4**. GO and KEGG classifications of DEGs for RNA-seq data. (a) GO classification of the unigenes of IFN-γtreated 5th generation (N05) of BMECs compared to control (NC). Red represents upregulated genes and green represents downregulated genes. The left and right y-axes denote the percent and number of genes in the category, respectively. (b) Pathway impact analysis showed the top 20 pathways of IFN-γ-treated 5th generation (N05) of BMECs compared to control (NC). The enriched pathways were represented as circles according to their scores from enrichment (y-axis) and topology analyses (pathway impact, x-axis) using MetaboAnalyst 3.0. The size and color of each circle reflect the pathway impact values and p values, respectively.** Figure S5**. Original uncropped blot images for LAP3 and GAPDH expression in Fig. 2C. Lanes within two vertical blue lines were utilized in Fig. 2C. The membrane utalized for blotting was boxed in red retangle.** Figure S6**. Original uncropped blot images for LAP3 and GAPDH expression in Fig. 2I. Lanes within two vertical blue lines were utilized in Fig. 2I. The membrane utalized for blotting was boxed in red retangle.** Figure S7**. Original uncropped blot images for ASS1 and GAPDH expression in Fig. 3F. Lanes within two vertical blue lines were utilized in Fig. 3F. The membrane utalized for blotting was boxed in red retangle.** Figure S8**. Original uncropped blot images for LAP3, ASS1 and GAPDH expression in Fig. 3G. Lanes within two vertical blue lines were utilized in Fig. 3G. The membrane utalized for blotting was boxed in red retangle.** Figure S9**. Original uncropped blot images for LAP3, cyclin A1, cyclin D1, p27 and GAPDH expression in Fig. 4D. Lanes within two vertical blue lines were utilized in Fig. 4D. The membrane utalized for blotting was boxed in red retangle.** Figure S10**. Original uncropped blot images for LAP3, cyclin A1, cyclin D1, p27 and GAPDH expression in Fig. 4F. Lanes within two vertical blue lines were utilized in Fig. 4F. The membrane utalized for blotting was boxed in red retangle.** Figure S11**. Original uncropped blot images for LAP3, HDAC2 and GAPDH expression in Figs. 5A and 6A. Lanes within two vertical blue lines were utilized in Figs. 5A and 6A. The membrane utalized for blotting was boxed in red retangle.** Figure S12**. Original uncropped blot images for LAP3, HDAC2 and GAPDH expression in Fig. 5B. Lanes within two vertical blue lines were utilized in Fig. 5B. The membrane utalized for blotting was boxed in red retangle.** Figure S13**. Original uncropped blot images for LAP3, HDAC2 and GAPDH expression in Fig. 5C. Lanes within two vertical blue lines were utilized in Fig. 5C. The membrane utalized for blotting was boxed in red retangle.** Figure S14**. Original uncropped blot images for HDAC2, cyclin A1, cyclin D1, p27 and GAPDH expression in Fig. 5D. Lanes within two vertical blue lines were utilized in Fig. 5D. The membrane utalized for blotting was boxed in red retangle.** Figure S15**. Original uncropped blot images for cyclin A1, cyclin D1, p27 and GAPDH expression in Fig. 6B. Lanes within two vertical blue lines were utilized in Fig. 6D. The membrane utalized for blotting was boxed in red retangle.** Figure S16**. Original uncropped blot images for LAP3, p-p38, p38 and GAPDH expression in Fig. 7A. Lanes within two vertical blue lines were utilized in Fig. 7A. The membrane utalized for blotting was boxed in red retangle.** Figure S17**. Original uncropped blot images for LAP3, p-ERK, ERK and GAPDH expression in Fig. 7D. Lanes within two vertical blue lines were utilized in Fig. 7A. The membrane utalized for blotting was boxed in red rectangle.

**Additional file 2: Table S1.** List of primers for qRT-PCR in the study.

## Data Availability

The RNA-seq data used in this study was openly available in figshare (10.6084/m9.figshare.19401290.v1). All other data supporting the conclusions of this article is included within the article and its additional file.

## Abbreviations

BMECs: Bovine mammary epithelial cells
LAP3: Leucine aminopeptidase 3
ASS1: Argininosuccinate synthetase
HDAC2: Histone deacetylase 2
NK: Natural killer cells
DEGs: Differentially expressed genes
HPLC–MS/MS: High performance liquid chromatography-mass spectrometry
Th1: T helper cell type 1
LAPs: Leucine aminopeptidase
VPA: Valproic acid
CCK-8: Cell counting kit-8
GO: Gene ontology
KEGG: Kyoto Encyclopedia of Genes and Genomes
qRT-PCR: Quantitative real-time polymerase-chain reaction
FBS: Fetal bovine serum
