# Supplementary material for: LAP3 contributes to IFN-γ-induced arginine depletion and malignant transformation of bovine mammary epithelial cells
Source: BMC Cancer. 2022 Aug 8;22:864. doi: 10.1186/s12885-022-09963-w (PMC9358085; doi:10.1186/s12885-022-09963-w)
Supplement: Supplementary file 1 — Additional file 1: Figure S1. Targeted metabolomics for internal standards. All the internal standards were well separated without interfering peaks. Figure S2. The levels of arginine, ornithine, citrulline in BMECs upon IFN-γ treatment using targeted metabolomics. BMECs were treatment with 10 ng/mL IFN-γ for 24 h. Targeted metabolomics was used to detect the changes of arginine (a), citrulline (b), and ornithine (c) upon IFN-γ treatment. Bars represent mean values with error bars to represent SD from twelve independent replicates. Differences between mean values were assessed by two-tailed Student’s t-test. *p < 0.05; **p < 0.01; ***p < 0.001, compared to control. Figure S3. qRT-PCR validation of RNA-seq data (a) and volcano plots for the DEGs (b). (a) 19 DEGs from the RNA-seq data were selected for qRT-PCR analysis. (b) Left, a volcano plot showing RNA-seq results of IFN-γ-treated 5th generation of BMECs (N05) to the control (no IFN-γ treatment, NC). Downregulated or upregulated genes were divided by |log2Ratio| ≥1 with false discovery rate (FDR) ≤ 0.01. Red dots for upregulated genes and blue dots for downregulated genes. Right, depiction of selected 10 most significantly changed genes. Figure S4. GO and KEGG classifications of DEGs for RNA-seq data. (a) GO classification of the unigenes of IFN-γtreated 5th generation (N05) of BMECs compared to control (NC). Red represents upregulated genes and green represents downregulated genes. The left and right y-axes denote the percent and number of genes in the category, respectively. (b) Pathway impact analysis showed the top 20 pathways of IFN-γ-treated 5th generation (N05) of BMECs compared to control (NC). The enriched pathways were represented as circles according to their scores from enrichment (y-axis) and topology analyses (pathway impact, x-axis) using MetaboAnalyst 3.0. The size and color of each circle reflect the pathway impact values and p values, respectively. Figure S5. Original uncropped blot images for LA [file 12885_2022_9963_MOESM1_ESM.pdf]

Supplementary Figures

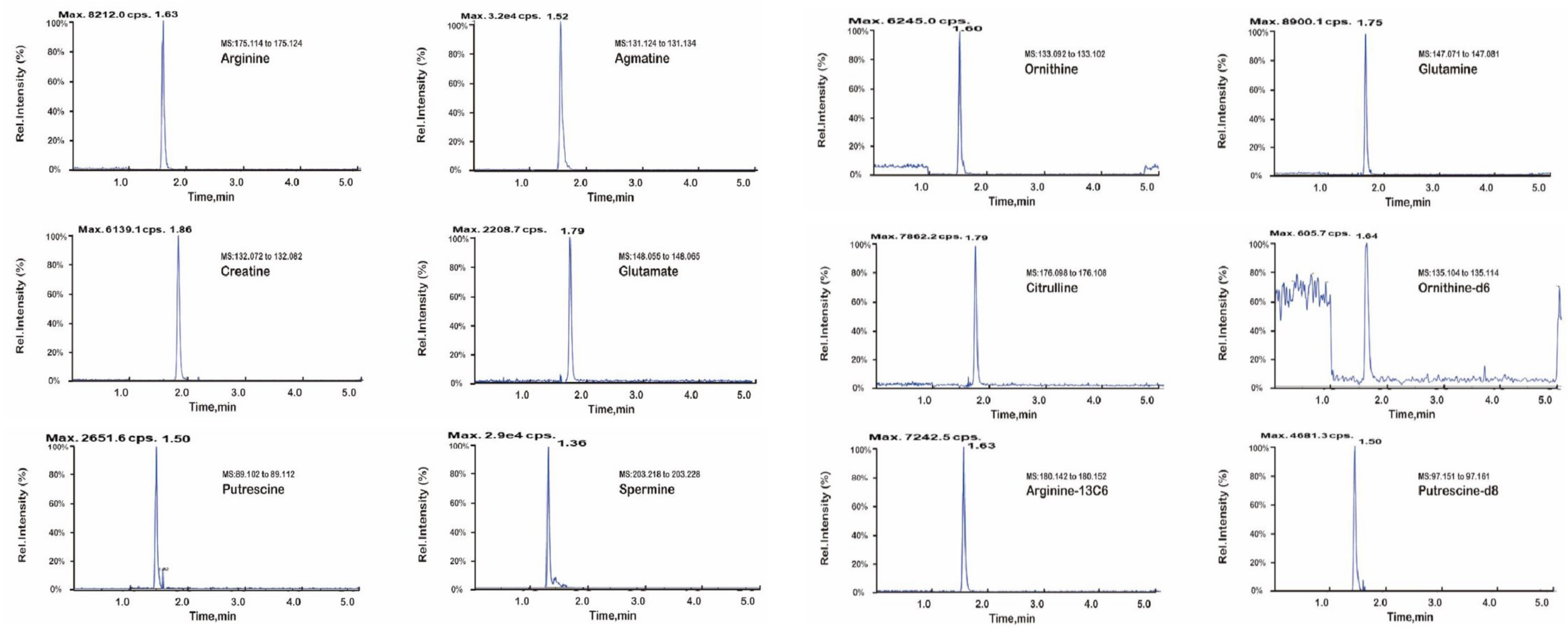

Figure S1. Targeted metabolomics for internal standards. All the internal standards were well separated without interfering peaks.

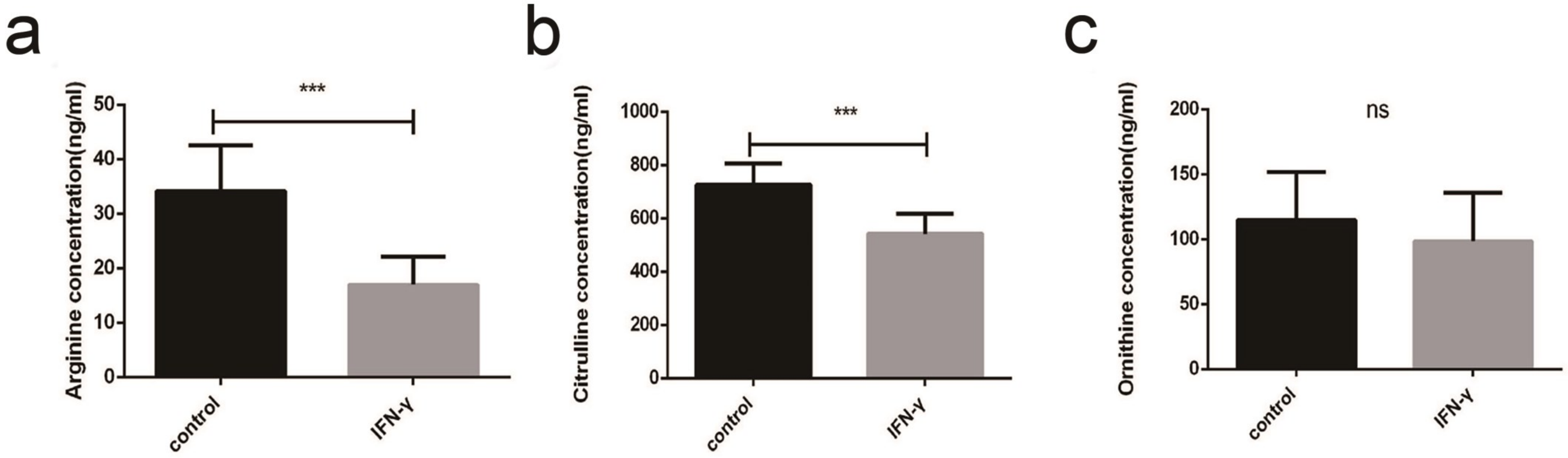

Figure S2. The levels of arginine, ornithine, citrulline in BMECs upon IFN-γ treatment using targeted metabolomics. BMECs were treatment with 10 ng/mL IFN-γ for 24 h. Targeted metabolomics was used to detect the changes of arginine (**a**), ornithine (**b**), and citrulline (**c**) upon IFN-γ treatment. Bars represent mean values with error bars to represent SD from twelve independent replicates. Differences between mean values were assessed by two-tailed Student's t-test. *p < 0.05; **p < 0.01; ***p < 0.001, compared to control.

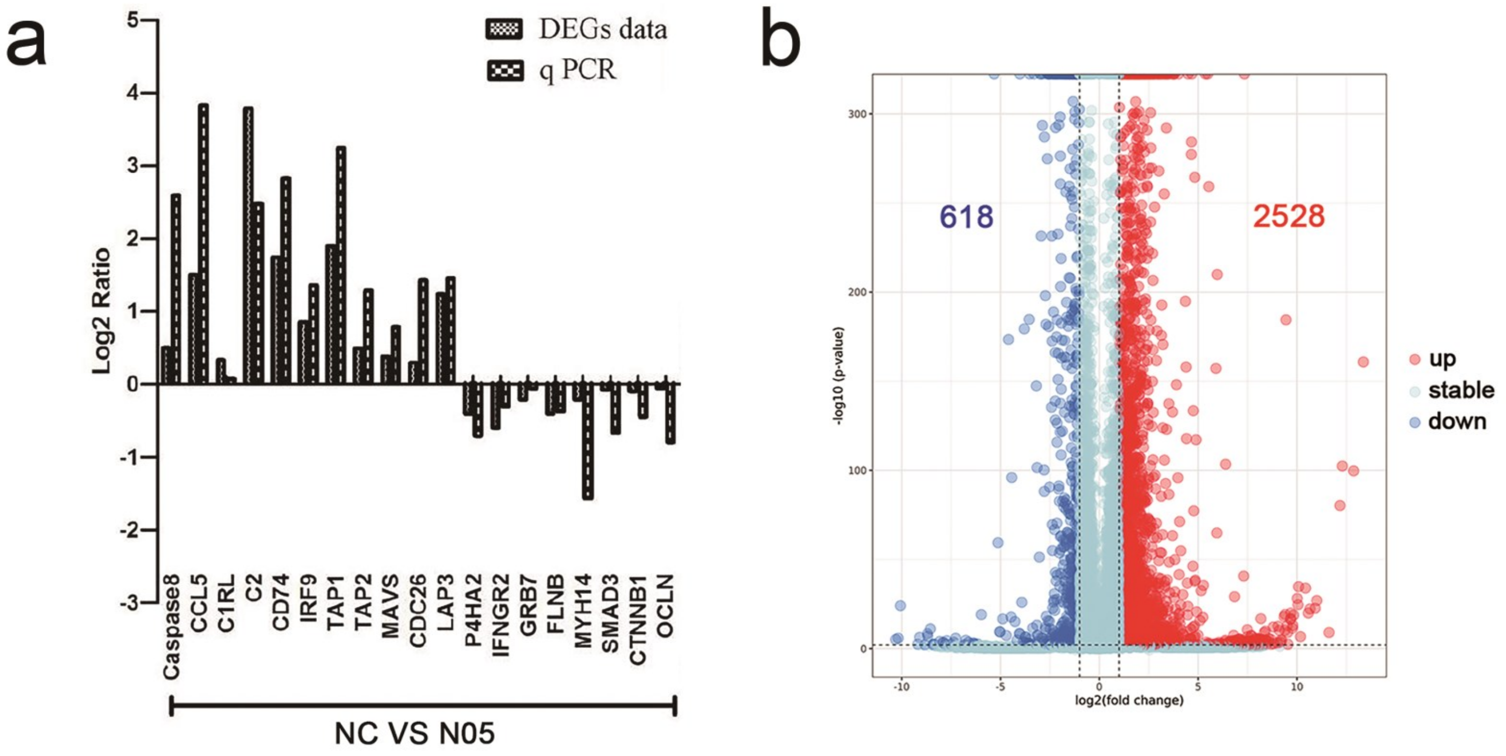

Gene	-log10p	log2FC
MAOB	11.9	6.87
NOS2	260	5.64
ALPL	25.2	3.39
LAP3	248	1.24
GATM	53.3	1.05
P4HA1	117	1.05
PYCR1	267	-1.19
ASL	148	-1.19
ARG2	147	-1.17
ASS1	142	-0.37

Figure S3. qRT-PCR validation of RNA-seq data (**a**) and volcano plots for the DEGs (**b**). (**a**) 19 DEGs from the RNA-seq data were selected for qRT-PCR analysis. (**b**) Left, a volcano plot showing RNA-seq results of IFN-γ-treated 5th generation of BMECs (N05) to the control (no IFN-γ treatment, NC). Downregulated or upregulated genes were divided by |log2Ratio| ≥1 with false discovery rate (FDR) ≤ 0.01. Red dots for upregulated genes and blue dots for downregulated genes. Right, depiction of selected 10 most significantly changed genes.

a

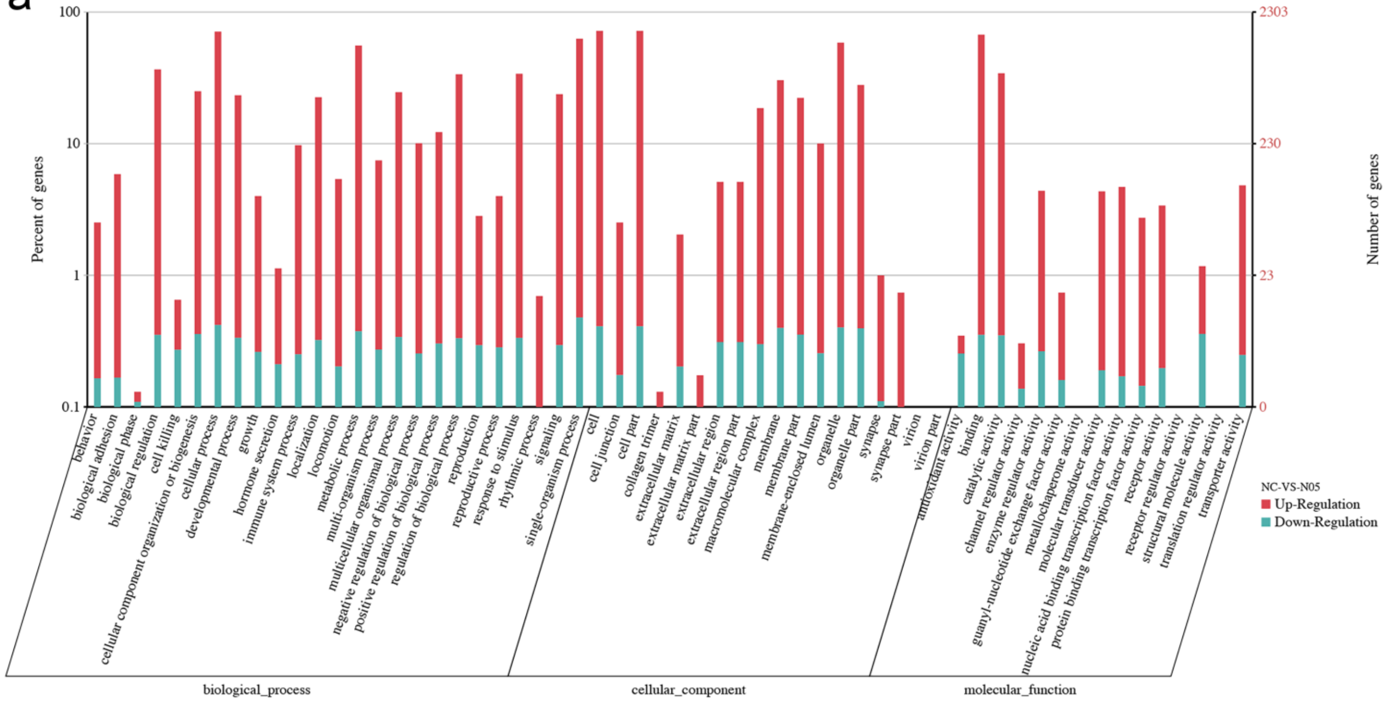

b

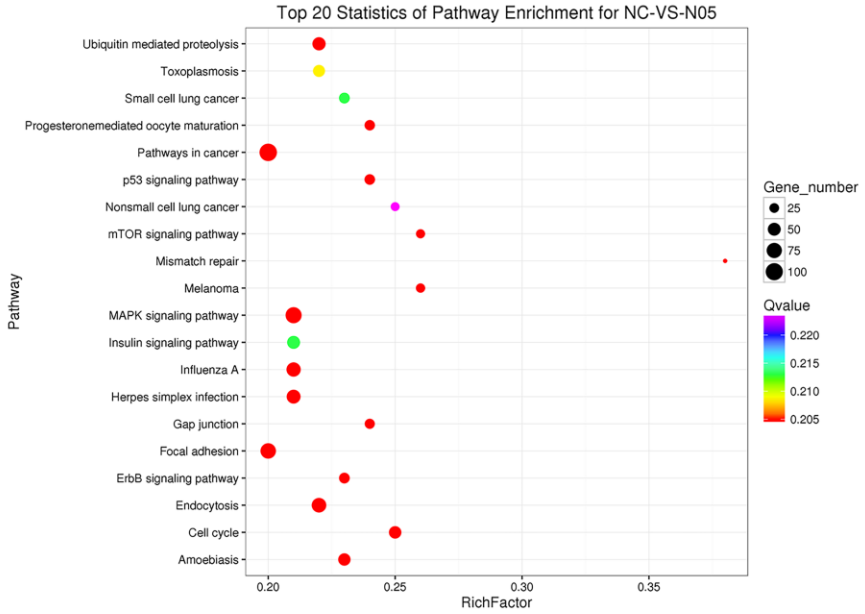

Figure S4. GO and KEGG classifications of DEGs for RNA-seq data. (**a**) GO classification of the unigenes of IFN-γ-treated 5th generation (N05) of BMECs compared to control (NC). Red represents upregulated genes and green represents downregulated genes. The left and right y-axes denote the percent and number of genes in the category, respectively. (**b**) Pathway impact analysis showed the top 20 pathways of IFN-γ-treated 5th generation (N05) of BMECs compared to control (NC). The enriched pathways were represented as circles according to their scores from enrichment (y-axis) and topology analyses (pathway impact, x-axis) using MetaboAnalyst 3.0. The size and color of each circle reflect the pathway impact values and p values, respectively.

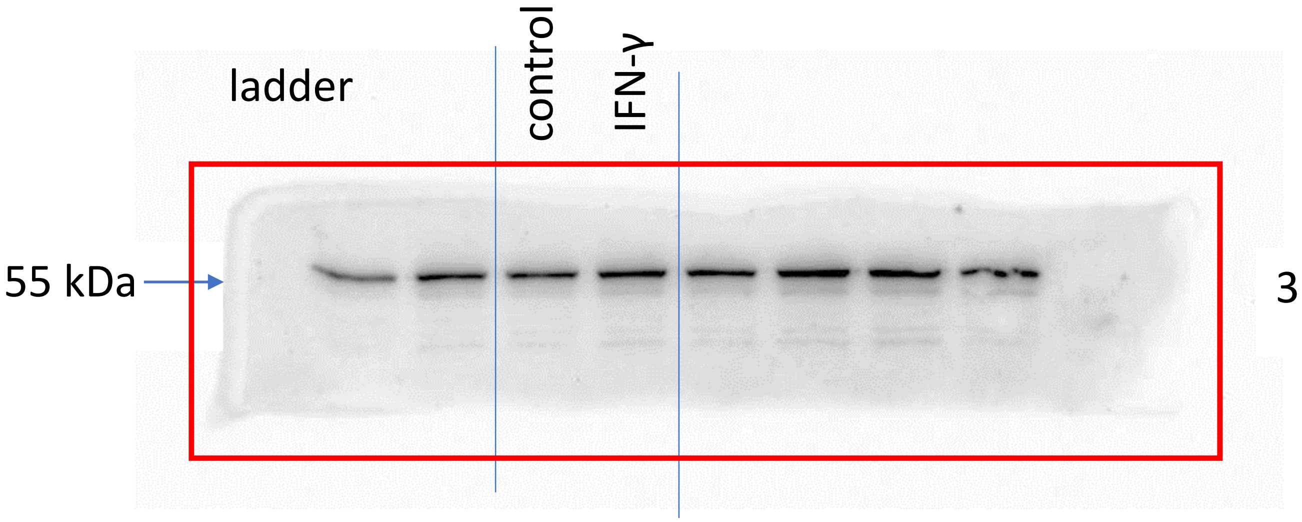

LAP3 (56 kDa)

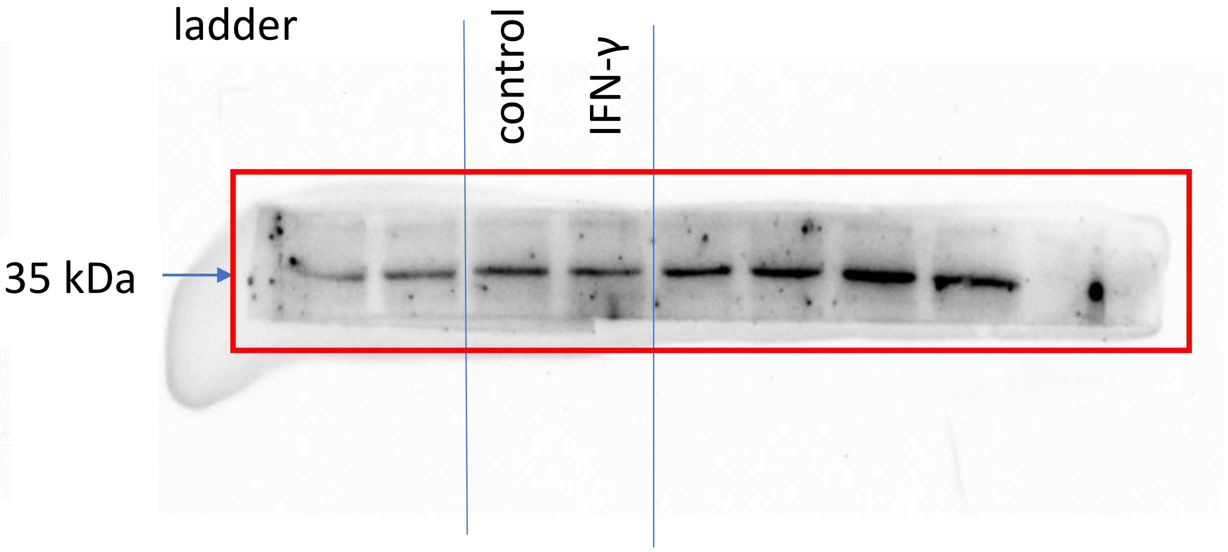

GAPDH (36 kDa)

Figure S5. Original uncropped blot images for LAP3 and GAPDH expression in Figure 2C. Lanes within two vertical blue lines were utilized in Figure 2C. The membrane utalized for blotting was boxed in red retangle.

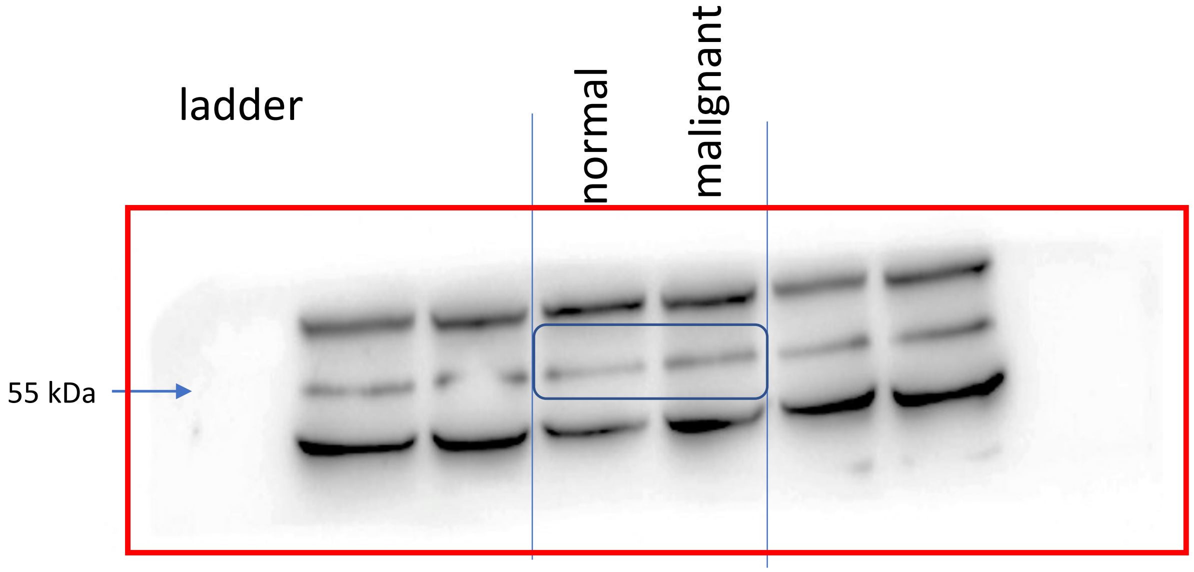

LAP3 (56 kDa), boxed

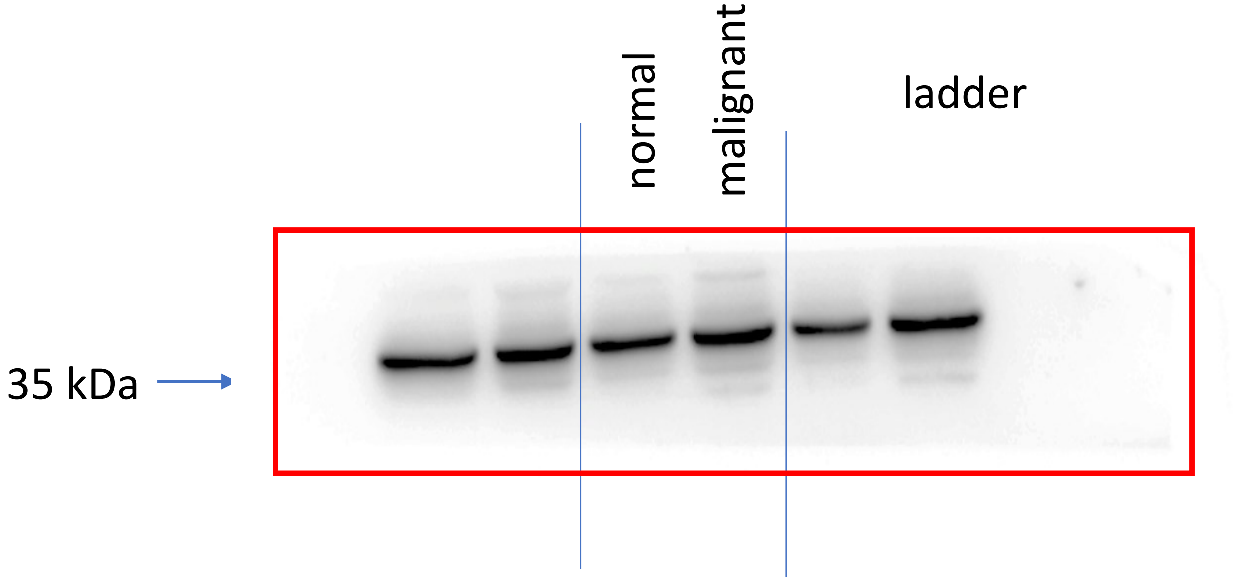

GAPDH, (36 kDa)

Figure S6. Original uncropped blot images for LAP3 and GAPDH expression in Figure 2I. Lanes within two vertical blue lines were utilized in Figure 2I. The membrane utalized for blotting was boxed in red retangle.

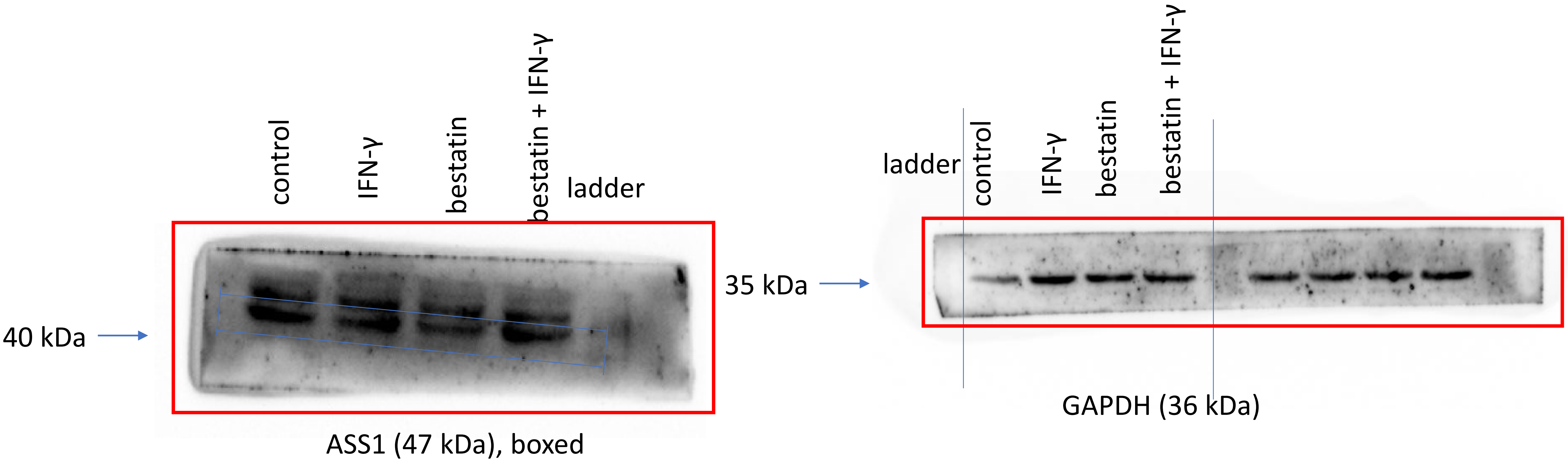

Figure S7. Original uncropped blot images for ASS1 and GAPDH expression in Figure 3F. Lanes within two vertical blue lines were utilized in Figure 3F. The membrane utalized for blotting was boxed in red retangle.

Figure S8. Original uncropped blot images for LAP3, ASS1 and GAPDH expression in Figure 3G.
Lanes within two vertical blue lines were utilized in Figure 3G. The membrane utalized for blotting was boxed in red retangle.

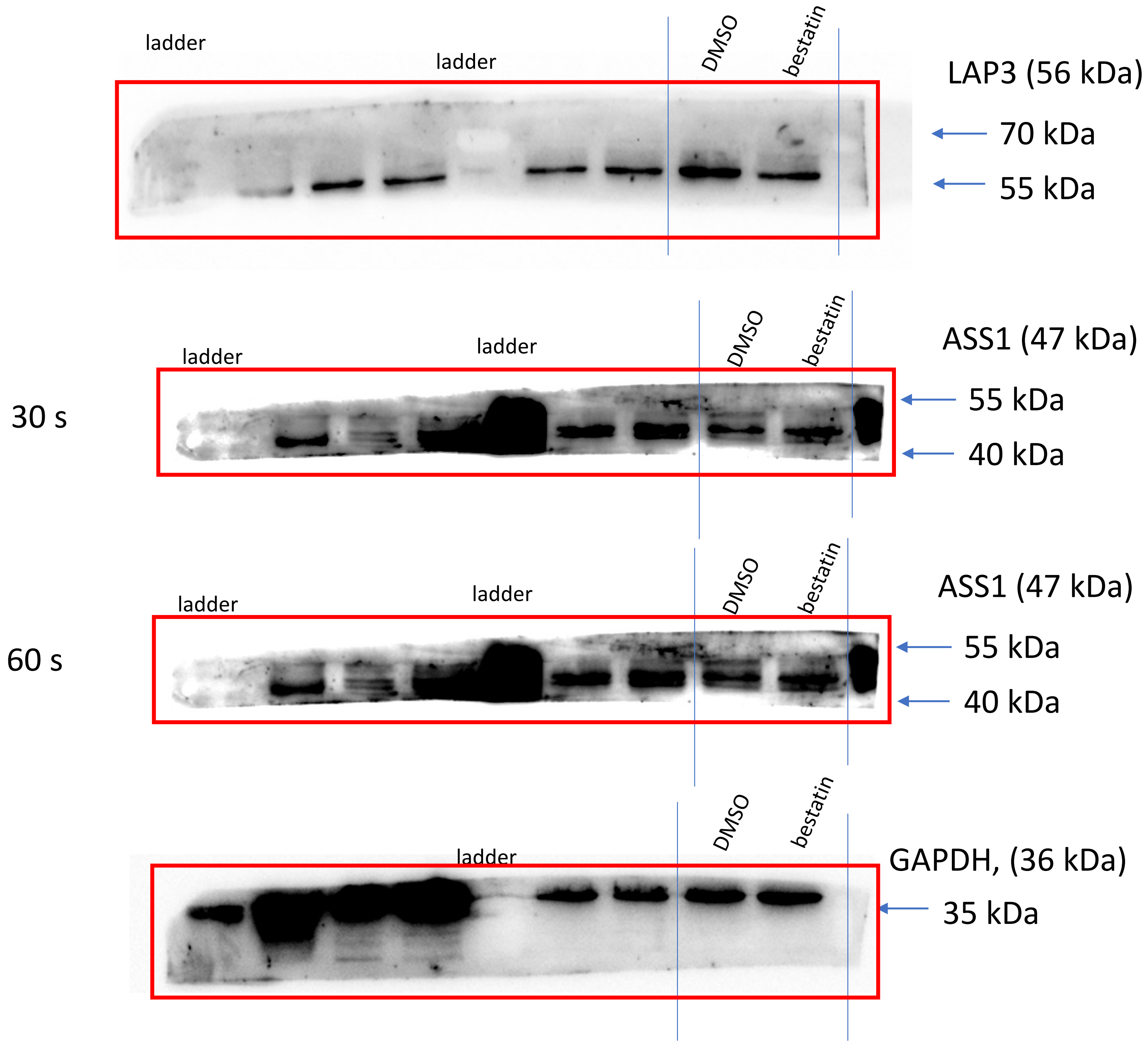

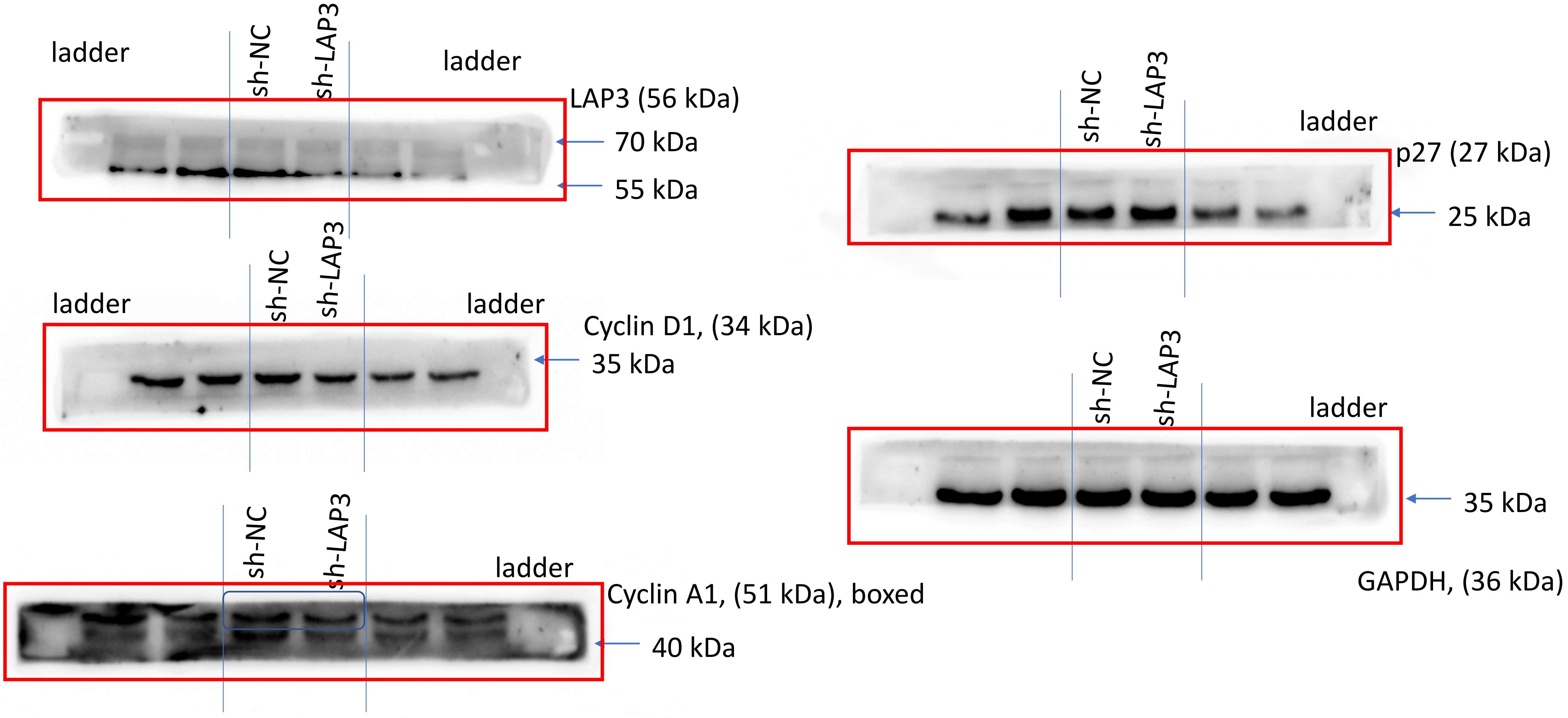

Figure S9. Original uncropped blot images for LAP3, cyclin A1, cyclin D1, p27 and GAPDH expression in Figure 4D. Lanes within two vertical blue lines were utilized in Figure 4D. The membrane utalized for blotting was boxed in red retangle.

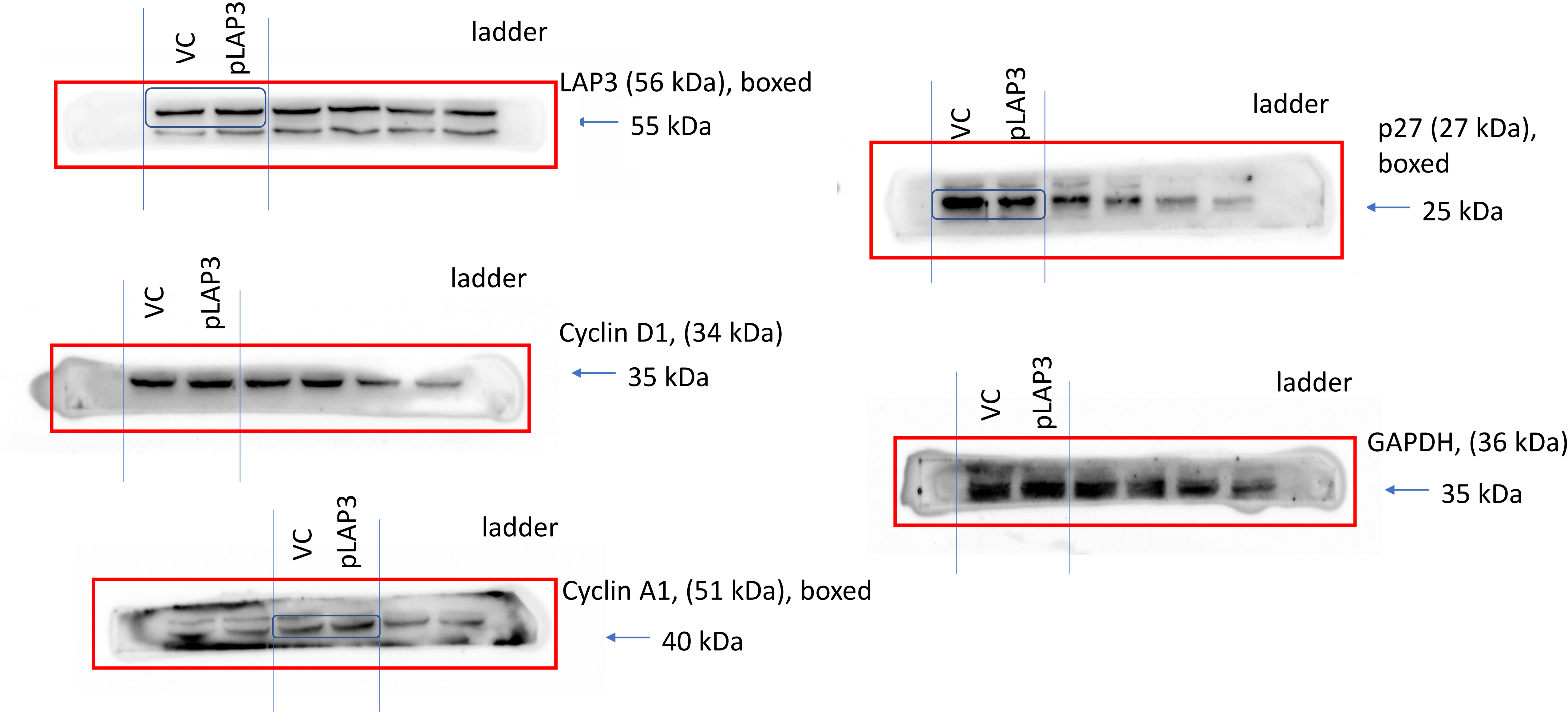

Figure S10. Original uncropped blot images for LAP3, cyclin A1, cyclin D1, p27 and GAPDH expression in Figure 4F. Lanes within two vertical blue lines were utilized in Figure 4F. The membrane utalized for blotting was boxed in red retangle.

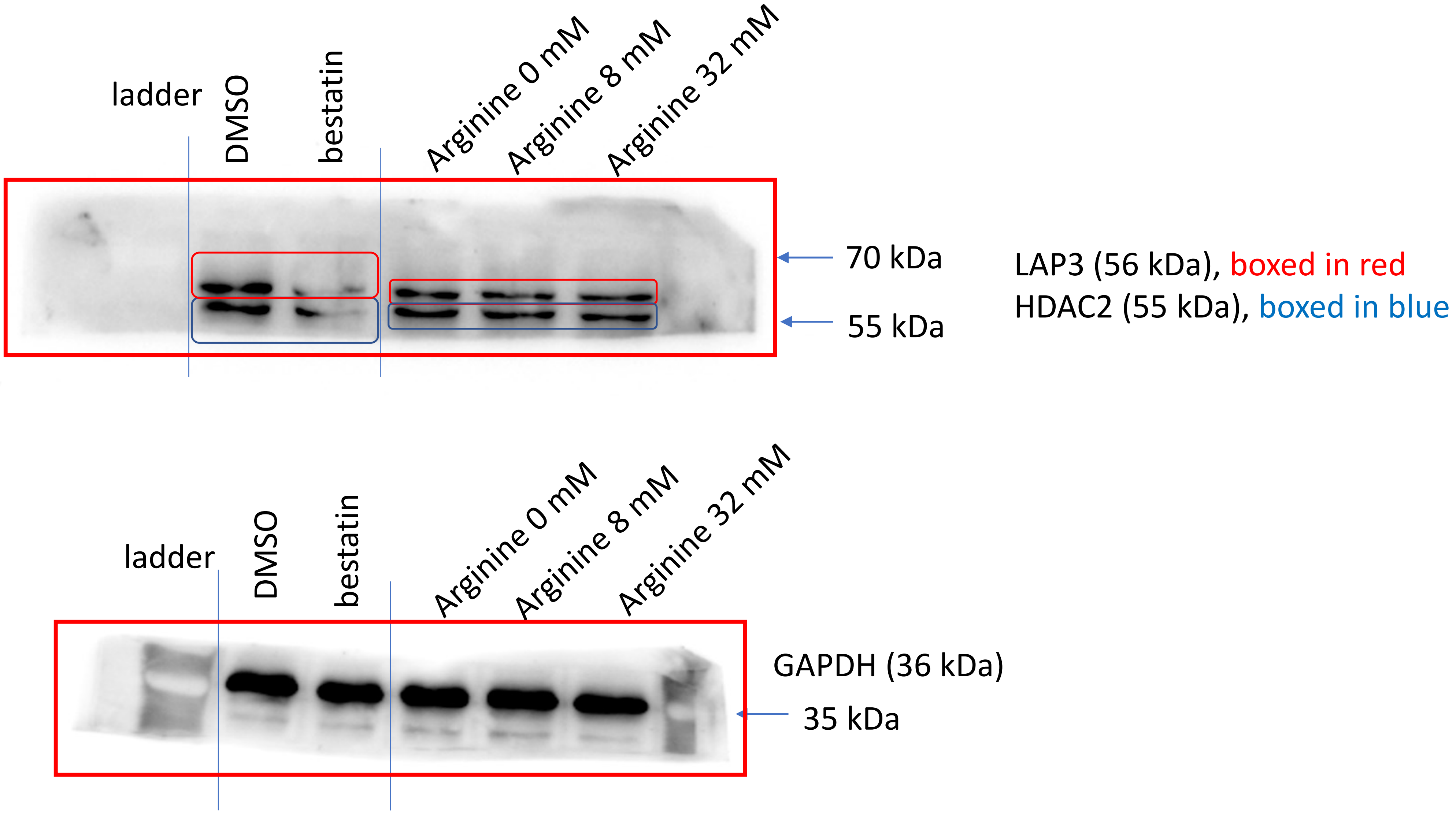

Figure S11. Original uncropped blot images for LAP3, HDAC2 and GAPDH expression in Figure 5A and 6A. Lanes within two vertical blue lines were utilized in Figure 5A and Figure 6A. The membrane utalized for blotting was boxed in red retangle.

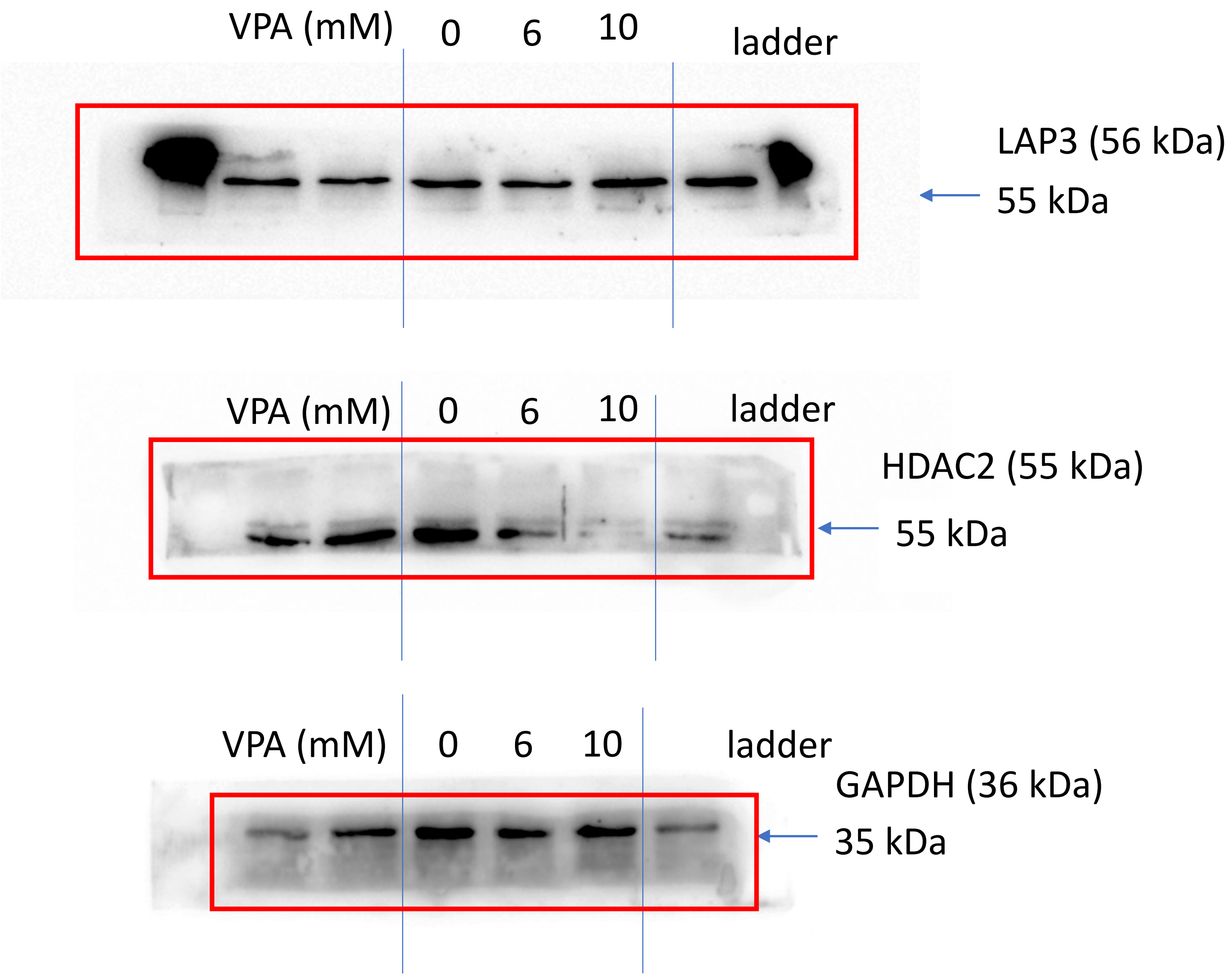

Figure S12. Original uncropped blot images for LAP3, HDAC2 and GAPDH expression in Figure 5B.
Lanes within two vertical blue lines were utilized in Figure 5B. The membrane utalized for blotting was boxed in red retangle.

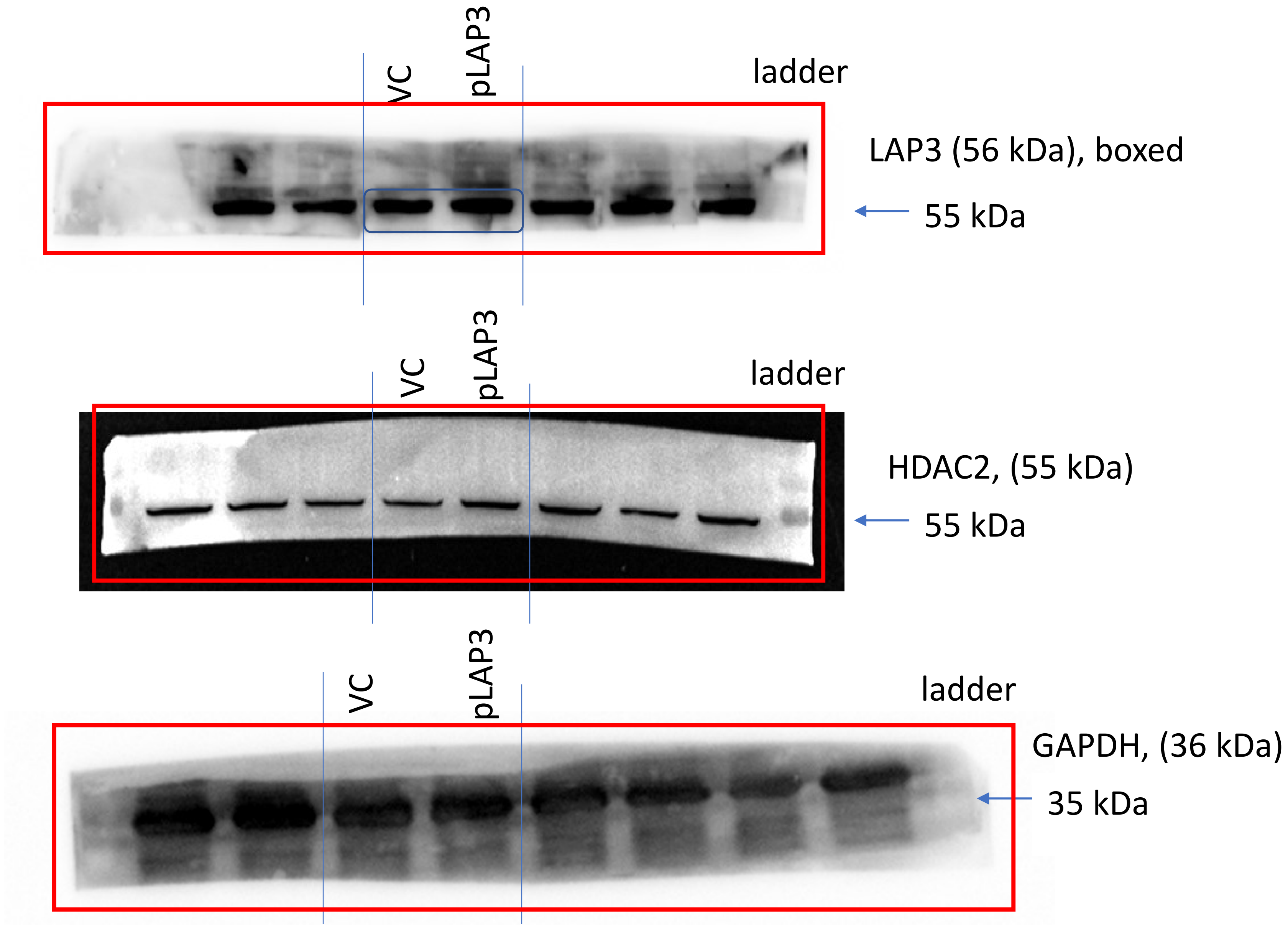

Figure S13. Original uncropped blot images for LAP3, HDAC2 and GAPDH expression in Figure 5C. Lanes within two vertical blue lines were utilized in Figure 5C. The membrane utalized for blotting was boxed in red retangle.

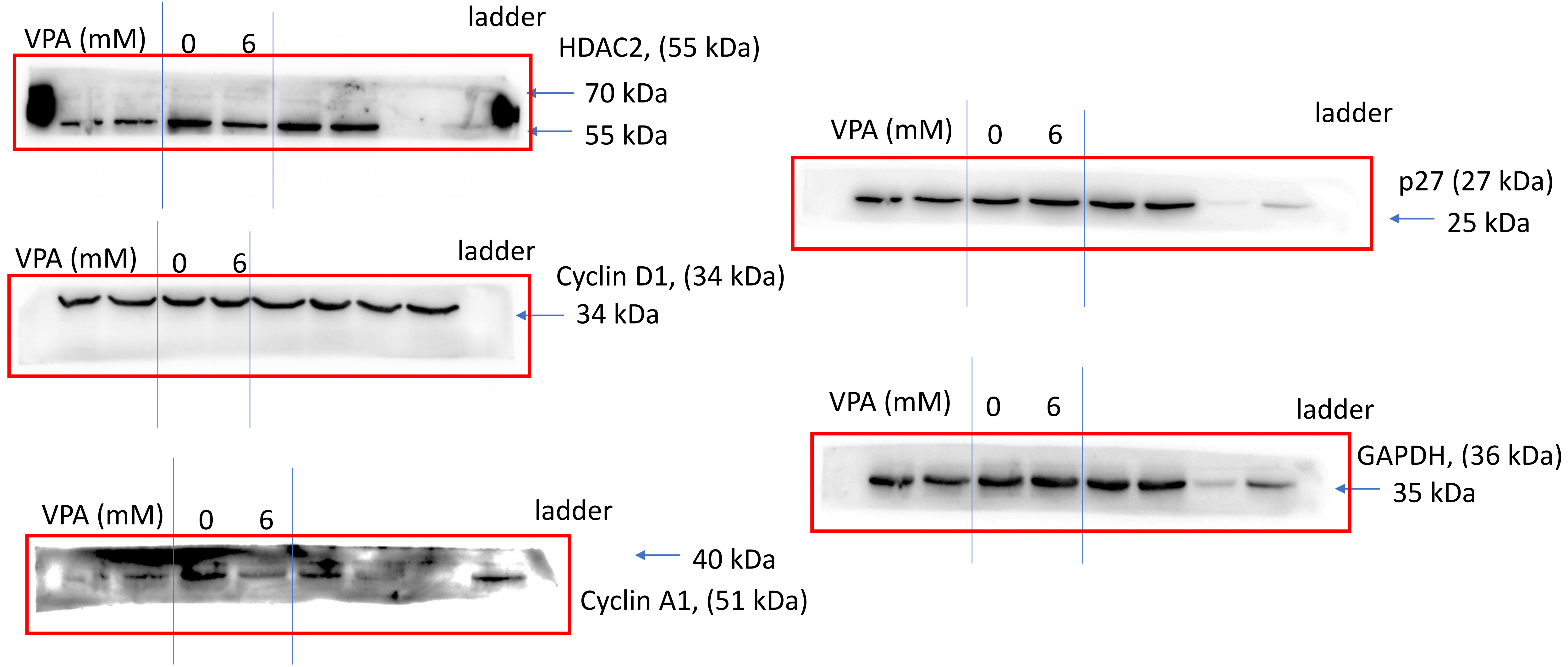

Figure S14. Original uncropped blot images for HDAC2, cyclin A1, cyclin D1, p27 and GAPDH expression in Figure 5D. Lanes within two vertical blue lines were utilized in Figure 5D. The membrane utalized for blotting was boxed in red retangle.

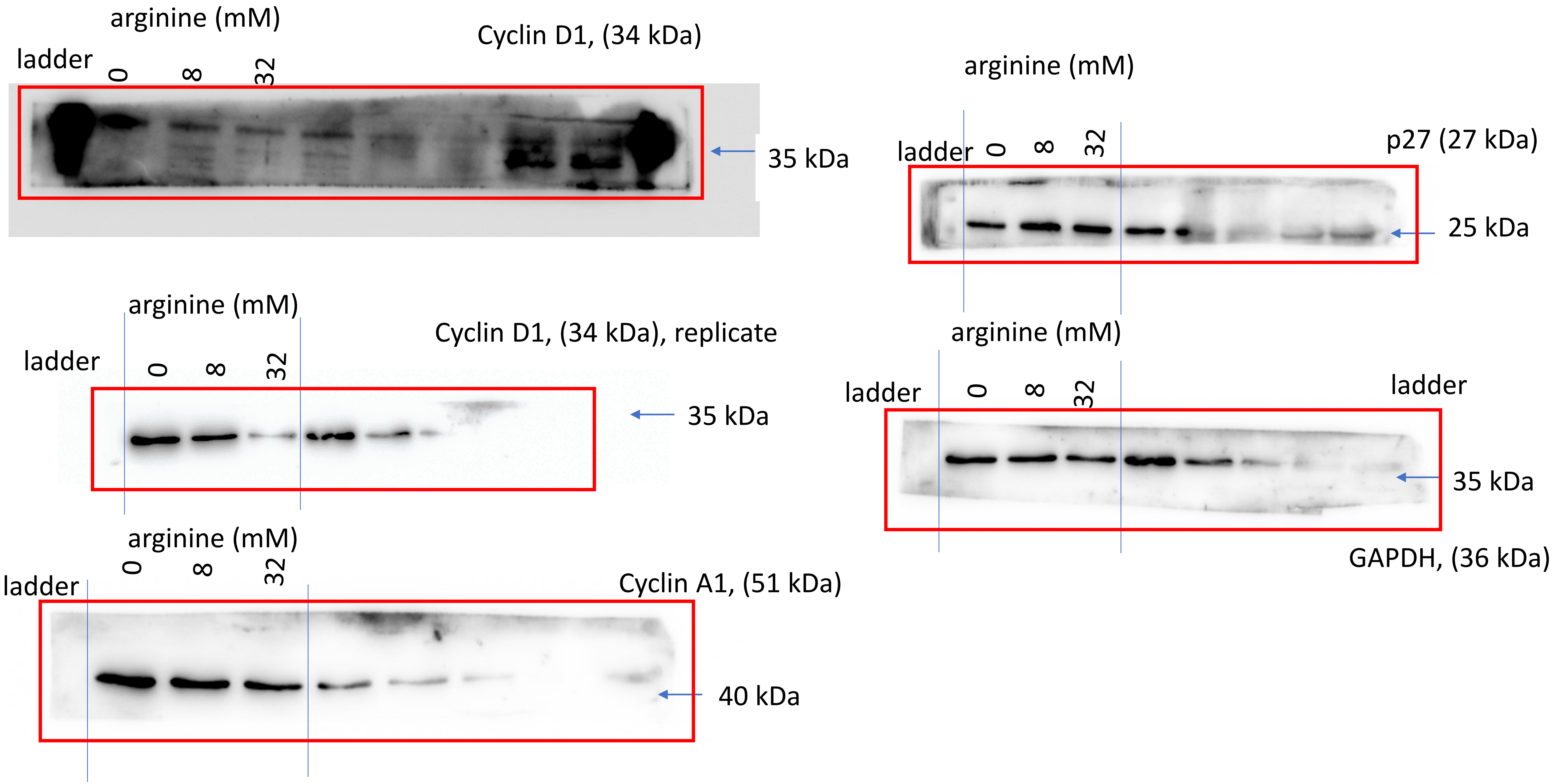

Figure S15. Original uncropped blot images for cyclin A1, cyclin D1, p27 and GAPDH expression in Figure 6B. Lanes within two vertical blue lines were utilized in Figure 6D. The membrane utalized for blotting was boxed in red retangle.

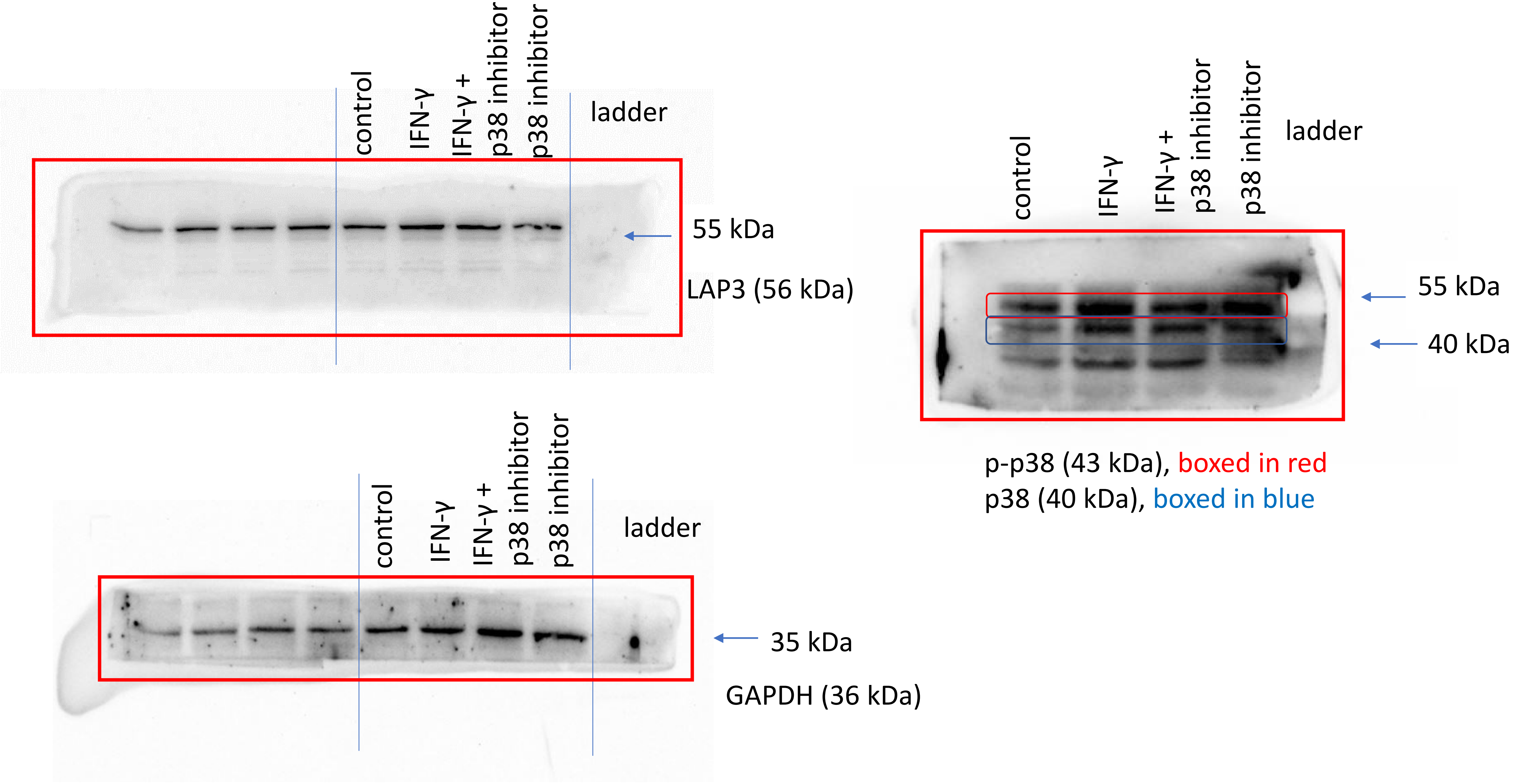

Figure S16. Original uncropped blot images for LAP3, p-p38, p38 and GAPDH expression in Figure 7A. Lanes within two vertical blue lines were utilized in Figure 7A. The membrane utalized for blotting was boxed in red retangle.

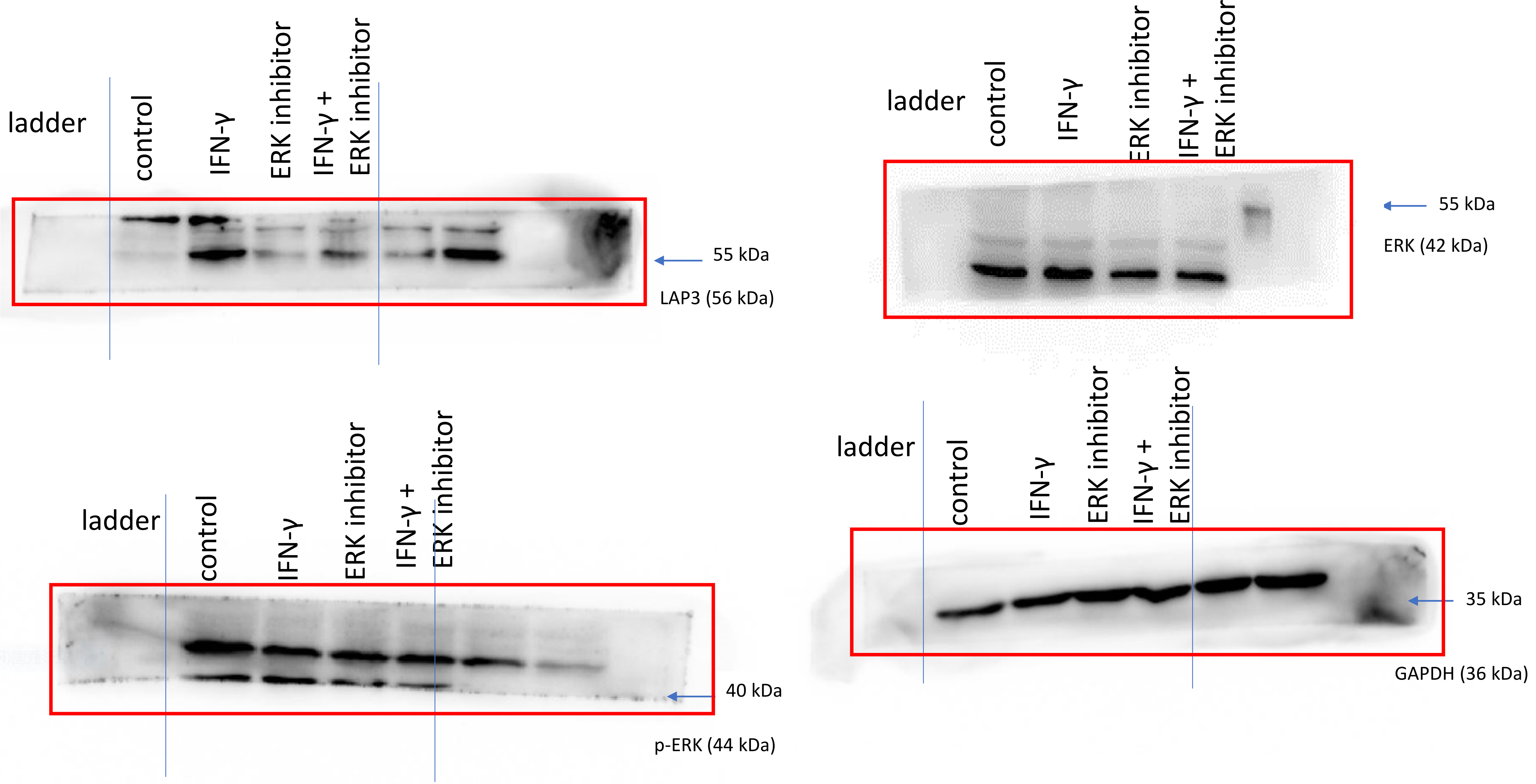

Figure S17. Original uncropped blot images for LAP3, p-ERK, ERK and GAPDH expression in Figure 7D. Lanes within two vertical blue lines were utilized in Figure 7A. The membrane utalized for blotting was boxed in red retangle.
