# Supplementary material for: LAP3 contributes to IFN-γ-induced arginine depletion and malignant transformation of bovine mammary epithelial cells
Source: BMC Cancer. 2022 Aug 8;22:864. doi: 10.1186/s12885-022-09963-w (PMC9358085; doi:10.1186/s12885-022-09963-w)
Supplement: Supplementary file 2 — Additional file 2: Table S1. List of primers for qRT-PCR in the study. [file 12885_2022_9963_MOESM2_ESM.docx]

## Supplementary tables

**Table S1** List of primers for qRT-PCR in the study.

| Gene Sense primer Antisense primers | | |
| --- | --- | --- |
| *LAP3* | AACATCGTAGGTTTGGCTCCTCTTTG  (26 bp) | CCGTTCCTGGCTCTAACAACATCC  (24 bp) |
| *β-actin* | TCCTGCGGCATTCACGAAACTAC (23 bp) | GTGTTGGCGTAGAGGTCCTTGC(22 bp) |

*LAP3* Gene ID: 781648  CDS bp 1560 bp pcr product size: 86 bp

*OTC*  Gene ID: 280979 CDS bp 1128 bp  pcr product size: 80 bp
